# Bioinformatics Analysis of Differentially Expressed Rhythm Genes in Liver Hepatocellular Carcinoma

**DOI:** 10.3389/fgene.2021.680528

**Published:** 2021-06-03

**Authors:** Huaifeng Liu, Yu Gao, Shangshang Hu, Zhengran Fan, Xianggang Wang, Shujing Li

**Affiliations:** ^1^School of Life Sciences, Bengbu Medical College, Bengbu, China; ^2^Anhui Province Key Laboratory of Immunology in Chronic Diseases, Bengbu Medical College, Bengbu, China; ^3^Research Center of Clinical Laboratory Science, School of Laboratory Medicine, Bengbu Medical College, Bengbu, China

**Keywords:** circadian rhythm, liver hepatocellular carcinoma, bioinformatics analysis, differentially expressed rhythm genes, chronotherapy

## Abstract

Liver Hepatocellular Carcinoma (LIHC), a malignant tumor with high incidence and mortality, is one of the most common cancers in the world. Multiple studies have found that the aberrant expression of rhythm genes is closely related to the occurrence of LIHC. This study aimed to use bioinformatics analysis to identify differentially expressed rhythm genes (DERGs) in LIHC. A total of 563 DERGs were found in LIHC, including 265 downregulated genes and 298 upregulated genes. KEGG pathway enrichment and GO analyses showed that DERGs were significantly enriched in rhythmic and metabolic processes. Survival analysis revealed that high expression levels of *CNK1D*, *CSNK1E*, and *NPA*S2 were significantly associated with the low survival rate in LIHC patients. Through cell experiment verification, the mRNA expression levels of *CSNK1D*, *CSNK1E*, and *NPAS2* were found to be strongly upregulated, which was consistent with the bioinformatics analysis of LIHC patient samples. A total of 23 nodes and 135 edges were involved in the protein–protein interaction network of *CSNK1D*, *CSNK1E*, and *NPAS2* genes. Clinical correlation analyses revealed that *CSNK1D*, *CSNK1E*, and *NPAS2* expression levels were high-risk factors and independently connected with the overall survival rate in LIHC patients. In conclusion, the identification of these DERGs contributes to the exploration of the molecular mechanisms of LIHC occurrence and development and may be used as diagnostic and prognostic biomarkers and molecular targets for chronotherapy in LIHC patients in the future.

## Introduction

Circadian rhythms, driven by endogenous circadian clocks, exist in almost all organisms on Earth. At the molecular level, circadian clocks are roughly similar in different organisms and consist of a series of transcription and translation feedback loops (Li and Zhang, 2015). In mammals, the transcription factors BMAL1 and CLOCK (CLK), forming heterodimers, drive *Period* (*Per*), and *Cryptochrome* (*Cry*) gene transcription through E-box elements in their promoter regions. A high degree of similarity to the mammalian system exists in *Drosophila*, where the transcription activators CYCLE (CYC) and CLOCK (CLK) heterodimerize and drive the transcription of *timeless* (*tim*) and *per* (Lowrey and Takahashi, 2011).

Disruption of the normal circadian rhythm has adverse effects on the physiology of mammals. Some clinical and laboratory experiments have shown that these disturbances can lead to various diseases, including mental illnesses, metabolic disorders, neurodegenerative diseases, and cancers (Gamble et al., 2014; Cash et al., 2015; Kiessling et al., 2017). A database related to circadian expression patterns of specific genes in clinical disorders including tumors has been published (Li et al., 2018). As one of the most common malignant tumors in the world, liver hepatocellular carcinoma (LIHC) ranks among the top 10 in terms of global mortality rate and is also a highly prevalent and harmful malignant tumor in China (Bray et al., 2018). However, the molecular mechanism underlying the carcinogenesis or progression of liver cancer is still unclear.

In the current study, we aim to identify differentially expressed rhythm genes (DERGs) through comprehensive bioinformatics analysis, which maybe benefit to explore the potential molecular mechanism underlying the occurrence and development of LIHC and may be used as diagnostic and prognostic biomarkers and molecular targets for chronotherapy in LIHC patients in the future.

## Materials and Methods

**FIGURE 1:**
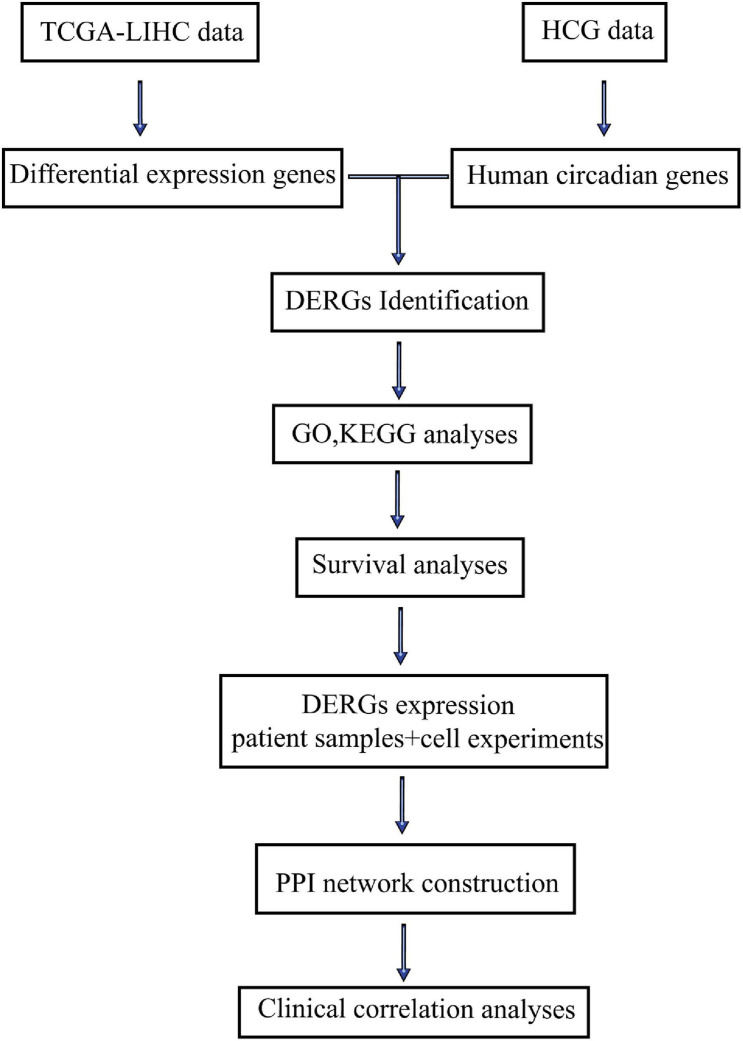
Flow diagram of this study.

The flow diagram of this study was shown in Figure 1. DERGs were found through The Cancer and Tumor Genome Atlas (TCGA) database and a human circadian genes database. GO function, KEGG pathway enrichment, protein–protein interaction (PPI) network and survival analyses of DERGs were performed using R software. DERGs expression levels were analyzed in LIHC patient samples and was verified in cell experiments. Further clinical correlation analyses were conducted to explore the value of DERGs in the timing treatment of LIHC.

### Data Collection

First, TCGA repository interface^1^ was entered. Then, TCGA-LIHC was selected in the Project column. Meanwhile, transcriptome profiling was selected in the Data Category column; Gene Expression Quantification was selected as the Data Type; RNA-seq was selected in the Experimental Strategy column; and HTSeq-FPKM was selected in the Workflow Type column. Finally, all the data that matched the above conditions were downloaded.

### Screening Differentially Expressed Genes

The differentially expressed genes (DEGs) between LIHC samples and non-neoplastic samples were screened using R software (version 4.0.2). DEGs were defined as those representing differences with *P* < 0.05. Using the Venny 2.1 online tool, 7168 DEGs were compared with the human circadian gene list (constructed by our research group) (Li et al., 2017). The intersection of both was taken; 563 DERGs were screened out and a volcano plot was drawn using R software (version 4.0.2).

### GO and KEGG Pathway Analysis

Gene Ontology (GO) analysis is a general method for large-scale functional enrichment research. Gene function includes biological process (BP), molecular function (MF), and cellular component (CC) (Harris et al., 2004). KEGG is a broad functional database that stores a wealth of data on biological pathways, genomes, diseases, and drugs (Ogata et al., 1999; Kanehisa et al., 2021). First, the DERGs’ official symbols were converted through the org.hs.eg.db package in R software (version 4.0.2); the cluster Profiler package was used for GO and KEGG pathway analyses; and GO plot package was used for cluster analysis. Significant differences were defined when both *P* and *Q* values were less than 0.05.

### Survival Analysis

The survival package of R software (version 4.0.2) was used to analyze *ARNTL*, *CSNK1D*, *CSNK1E*, *NPAS2*, *NR1D1*, *PER1*, *PER2*, *PER3*, *PRKAG2*, and *RORA* gene expression profiles and overall survival (OS) in TCGA-LIHC patients (excluding patent information with no survival time). Then, the survival curve analysis of the CSNK1D, CSNK1E, and NPAS2 genes was verified by using the KM-plotter online tool. On the main interface of the KM-plotter, “Start KM Plotter for liver cancer” in “RNA-seq” was selected; the gene name to be searched was inputted and the default values for other options were selected.

### Matching Analysis

After screening and sorting the patient samples, the tumor samples were paired with the normal samples from the same patients (a total of 50 pairs). In addition, the expression levels of the *CSNK1D*, *CSNK1E*, and *NPAS2* genes were extracted, and the matching graphs were drawn in R software (version 4.0.2).

### Cell Culture

WRL-68, SMMC-7721, SNU-449, and HUH-7 cells were cultured in DMEM (Gibco, Thermo Fisher Scientific, United States) with 10% fetal bovine serum (Life Technologies, United States) and 1% penicillin and streptomycin (100 U/ml penicillin and 100 μg/ml streptomycin, Life Technologies) at 37°C in a humidified incubator containing 5% CO_2_.

### RNA Extraction and RT-qPCR

Total RNA was extracted from cells using TRIzol (Invitrogen). Any contaminating genomic DNA was removed with RQ1 DNase (Promega, Madison, WI, United States) digestion, and the total RNA was directly amplified using the TransScript Green One-Step qRT-PCR SuperMix (TransGen Biotechnology, Beijing, China). All qPCRs were performed using the Step One Plus Real-Time PCR System (Life Technologies). The primers used for expression analysis were as follows: CSNK1D-fw: caaaaccaaacaccctcagc. CSNK1 D-rv: catcaccatgacgttgtagtcc. CSNK1E-fw: cgtgtggggaacaagtaccg. CSNK1E-rv: gatgttggcacccaggtagat. NPAS2-fw: cgtgttggaaa aggtcatcgg. NPAS2-rv: tccagtcttgctgaatgtcac.

### Protein–Protein Interaction Network Construction

The website of Search Tool for the Retrieval of Interacting Genes Database (STRING)^2^ was a biological database designed to construct a PPI network of DERGs based on the known and predicted PPIs and then analyze the functional interactions between proteins. Subsequently, the PPI network was visualized by means of Cytoscape software (version 3.7.2).

### Clinical Correlation Analyses

The relevant clinical data of the hepatoma patients, namely the phenotypes of TCGA LIHC (*n* = 377), were downloaded from TCGA database. In addition, according to the clinical characteristics of patients (such as age, sex, and tumor stage), we excluded some patients whose clinical data were incomplete; ultimately, we obtained data from 235 patients for Cox analysis to assess the combined effects of clinical characteristics and gene expression.

### Statistical Analysis

Statistical tests were performed using R software (version 4.0.2). The *t*-test or Mann–Whitney test was used for comparisons between the two groups. Schoenfeld individual tests were used to evaluate the proportional hazards assumption for the fit of the Cox regression model. We used univariate and multivariate Cox proportional hazards models to evaluate the survival analysis. The optimal cut-off value generated by the “surv cutpoint” function of R package was used for survival analysis, and expression levels were divided by dichotomy. In addition, statistical significance in cell line experiments was assessed in *t* tests in GraphPad Prism version 8 software. A value of *P* < 0.05 was considered statistically significant (^∗^*P* < 0.05, ^∗∗^*P* < 0.01, ^∗∗∗^*P* < 0.001).

## Results

### Identification of DERGs

**FIGURE 2:**
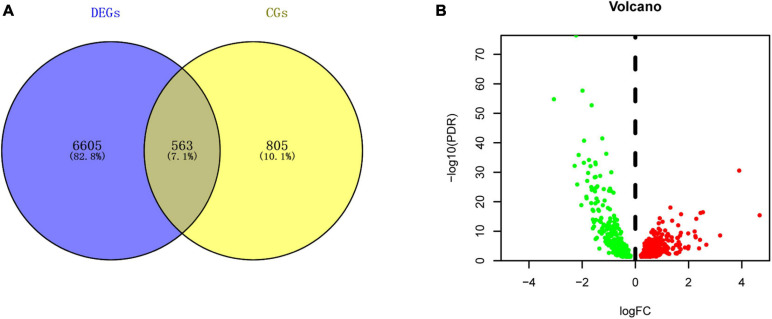
Identification of DERGs. **(A)** Intersection map of genes in the LIHC and CGDB datasets. **(B)** Volcano plot of DERGs. The green and red dots represent the downregulated and upregulated genes, respectively.

A total of 7168 DEGs and 1368 circadian genes were identified in the analyses of the LIHC-FPKM and CGDB datasets, respectively. Among them, 563 DERGs were found in both datasets (Figure 2A and Supplementary Table 1). Compared with normal liver tissue samples, 265 DERGs were downregulated and 298 DERGs were upregulated in LIHC tissue samples (Figure 2B).

### GO Analysis of DERGs

**FIGURE 3:**
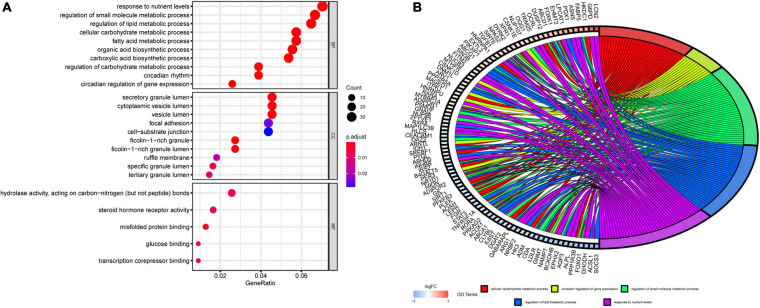
GO enrichment analysis of DERGs. **(A)** Biological process, cell components, and molecular function enrichment analyses of DERGs. **(B)** GO cluster analyses.

To further evaluate the functions and mechanisms of these DERGs, GO enrichment analysis of BPs indicated that the DERGs were mainly enriched in cellular carbohydrate metabolic process, circadian regulation of gene expression, regulation of small molecule metabolic process, regulation of lipid metabolic process, and response to nutrient levels and other processes. GO enrichment analysis of CCs indicated that the DERGs were mainly enriched in vesicle lumen and granule lumen. GO enrichment analysis of MFs indicated that the DERGs were mainly enriched in hydrolase activity, acting on carbon nitrogen bonds, steroid hormone receptor activity, and so on (Figure 3A). To further analyze the enrichment results, we performed gene cluster analysis on the BP modules with the top *P*-values. The results revealed that DERGs were significantly associated with rhythmic and metabolic processes (Figure 3B).

### KEGG Pathway Analysis of DERGs

**FIGURE 4:**
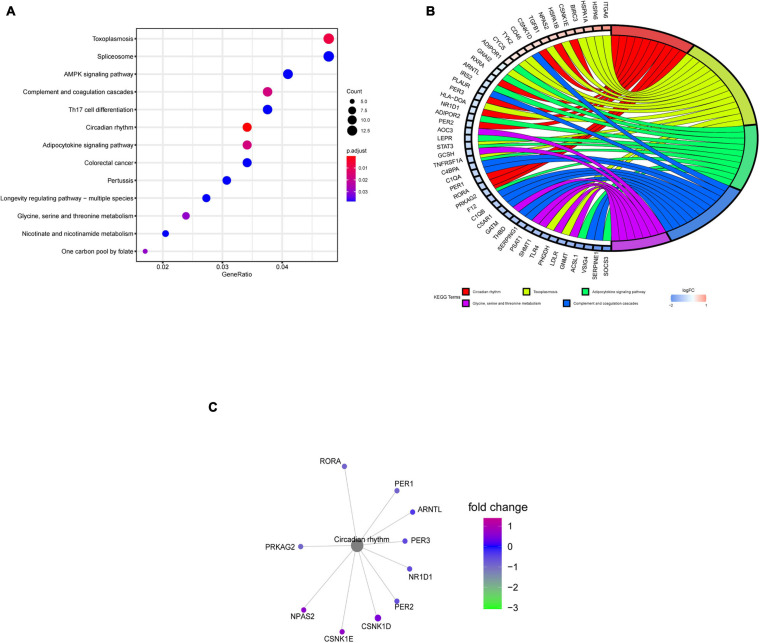
KEGG pathway enrichment analysis of DERGs. **(A)** KEGG pathway analysis of DERGs. **(B)** KEGG pathway cluster analyses. **(C)** Significantly enriched genes in the circadian rhythm pathway.

The KEGG pathway enrichment analysis indicated that the circadian rhythm pathway, the adipocytokine signaling pathway, and the glycine, serine, and threonine metabolism pathways were significantly affected, especially the circadian rhythm pathway, with *P* < 0.001 (Figure 4A). Gene cluster analysis of the KEGG pathways showed that DERGs were also significantly associated with rhythmic and metabolic processes (Figure 4B). In particular, the *CNK1D*, *CSNK1E*, *NPAS2*, *PER1*, *PER2*, *PER3*, *ARNTL*, *NR1D1*, *PRKAG2*, and *RORA* genes were significantly enriched in the circadian rhythm pathway (Figure 4C).

### Survival Analysis

**FIGURE 5:**
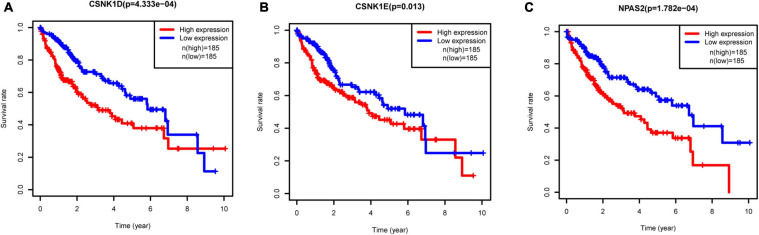
Relationships between three hub gene expression levels and survival rate in LIHC. **(A)** *CSNK1D*, **(B)** *CSNK1E*, and **(C)** *NPAS2*.

To investigate the prognostic values of DERGs, a total of 377 liver cancer samples were available for the analysis of OS (Supplementary Table 2). We found that higher expression levels of *CSNK1D, CSNK1E*, and *NPAS2* were significantly related to shorter OS of liver cancer patients (Figure 5). Conversely, the expression levels of *ARNTL*, *NR1D1*, *PER1*, *PER2*, *PER3*, *PRKAG2*, and *RORA* were not significantly related to the OS of liver cancer patients (Supplementary Figure 1). KM-plotter online tool further validated these results (Supplementary Figure 2).

### Analysis of DERGs Expression Levels

**FIGURE 6:**
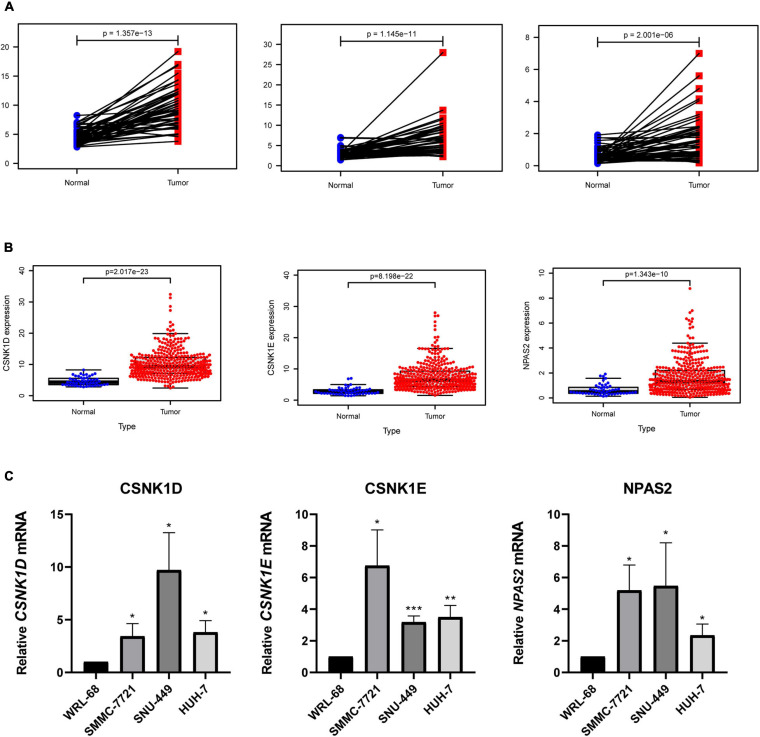
Expression levels of 3 hub genes in LIHC. **(A)** *CSNK1D*, *CSNK1E*, and *NPAS2* expression levels in normal and LIHC tissues. **(B)** *CSNK1D*, *CSNK1E*, and *NPAS2* expression levels in LIHC tissues and their matched controls. **(C)** The mRNA expression levels of *CSNK1D*, *CSNK1E*, and *NPAS2* in LIHC cell lines. The experiments were performed independently for three times. *P < 0.05, **P < 0.01, ***P < 0.001.

To explore the expression levels of genes (*CSNK1D*, *CSNK1E*, and *NPAS2)* in liver cancer, we compared the mRNA expression levels of tumor and normal tissues. The results revealed that the *CSNK1D*, *CSNK1E*, and *NPAS2* mRNA levels were strongly upregulated in LIHC samples (Figure 6A). The patient samples were then further analyzed and sorted (Supplementary Table 3), and the normal and tumor samples from the same patients were matched for comparisons (a total of 50 pairs) of their expression levels. We obtained the same results as before (Figure 6B). Additionally, in the liver cancer cell experiment, similar results were obtained, in which the *CSNK1D*, *CSNK1E*, and *NPAS2* mRNA expression levels were strongly upregulated compared with those in normal liver cells (Figure 6C).

### PPI Network Analysis

**FIGURE 7:**
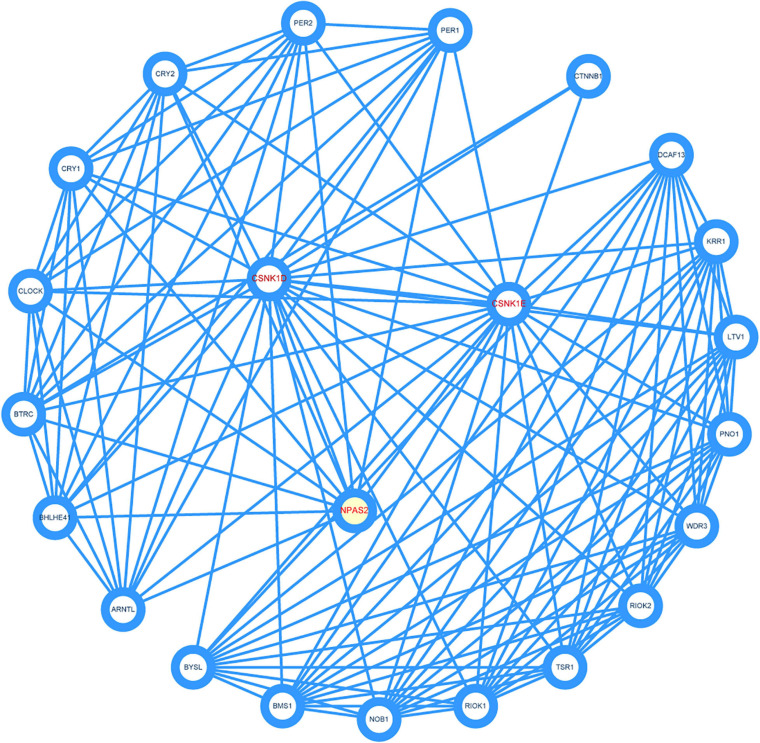
Construction of protein-protein interaction (PPI) network. Three hub genes were marked red.

To identify the key genes and important gene modules, the PPI network of CSNK1D, CSNK1E, and NPAS2 genes was constructed, consisting of 23 nodes and 135 edges (Figure 7). These interacting genes were *PER1*, *PER2*, *CRY1*, *CRY2*, *CLOCK*, *BTRC*, *BHLHE41*, *ARNTL*, *BYSL*, *BMS1*, *NOB1*, *RIOK1*, *TSR1*, *RIOK2*, *WDR3*, *PNO1*, *LTV1*, *KRR1, DCAF13*, and *CTNNB1*, which were mainly associated with circadian pathway, Hedgehog signaling pathway and Hippo signaling pathway.

### Correlations Between DERGs Expression and Clinicopathological Factors in LIHC

**FIGURE 8:**
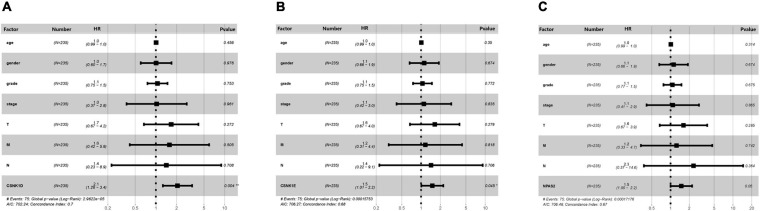
Multivariate Cox analysis of clinical characteristics. **(A)** CSNK1D, **(B)** CSNK1E, and **(C)** NPAS2. HR, hazard ratio; T, topography; N, lymph node; M, metastasis.

To further verify the clinical diagnostic value of the three DERGs, the univariate Cox analyses were performed to verify the prognostic role of DERGs in liver cancer. They show that CSNK1D expression (HR = 2.28; 95% CI = 1.47–3.54), CSNK1E expression (HR = 1.61; 95% CI = 1.12–2.33), NPAS2 expression (HR = 1.72; 95% CI = 1.18–2.51), TNM stage, and invasion depth were high risk factors (Supplementary Tables 4–6). Similarly, the results of multivariate analysis showed that CSNK1D (HR = 2.06; 95% CI = 1.26–3.37), CSNK1E (HR = 1.50; 95% CI = 1.01–2.22) and NPAS2 (HR = 1.49; 95% CI = 0.99–2.21) were also independently associated with OS (Figures 8A–C and Supplementary Table 7). Overall, the *CSNK1D*, *CSNK1E*, and *NPAS2* genes were adverse prognostic factors and independent prognostic markers.

## Discussion

Liver Hepatocellular Carcinoma is a common type of cancer that is associated with poor prognosis and high mortality (Yang and Roberts, 2010; Yang et al., 2019). Although research on the pathology of liver cancer has gradually deepened in the last few years and progress has been made in the management of diagnosis and treatment strategies for patients with liver cancer, the treatment success rate and clinical outcomes are still very poor (Lurje et al., 2019; Villanueva, 2019). With the continuous discovery of molecular biomarkers, prognostic indicators will become a promising clinical tool for liver cancer (Xu et al., 2018). Therefore, it is necessary to develop novel diagnostic and prognostic biomarkers for liver cancer to assist in clinical diagnosis and prediction.

Epidemiological studies have shown that the disruption of normal circadian rhythms may increase the risk of many types of cancer, such as breast, prostate, rectal, and endometrial cancers (Winter et al., 2007; Taniguchi et al., 2009; Kuo and Ladurner, 2019). In addition, abnormal expression levels of circadian clock genes are closely related to tumor development. Studies have found that mutations in BMAL1, CRY1, and CRY2 are often associated with a higher risk and recurrence of acute myeloid leukemia and endometrial, ovarian, colorectal, and breast cancers (Hasakova et al., 2018; Burgermeister et al., 2019; Kwon et al., 2020). *Per 1*, *Per 2*, and *Per 3* are frequently expressed in human breast and colorectal cancers, non-small-cell lung cancer (NSCLC), hepatocellular and cervical squamous cell carcinomas, and glioma (Yeh et al., 2005; Mostafaie et al., 2009; Lin et al., 2019). Studies have shown that clock genes can regulate the expression of proto-oncogenes and tumor suppressor genes and participate in the cell cycle, apoptosis, DNA damage, and other processes. These findings suggest that there is a network of mechanisms between rhythm genes and cancer and several DERGs can be used as prognostic markers for clinical diagnosis.

**FIGURE 9:**
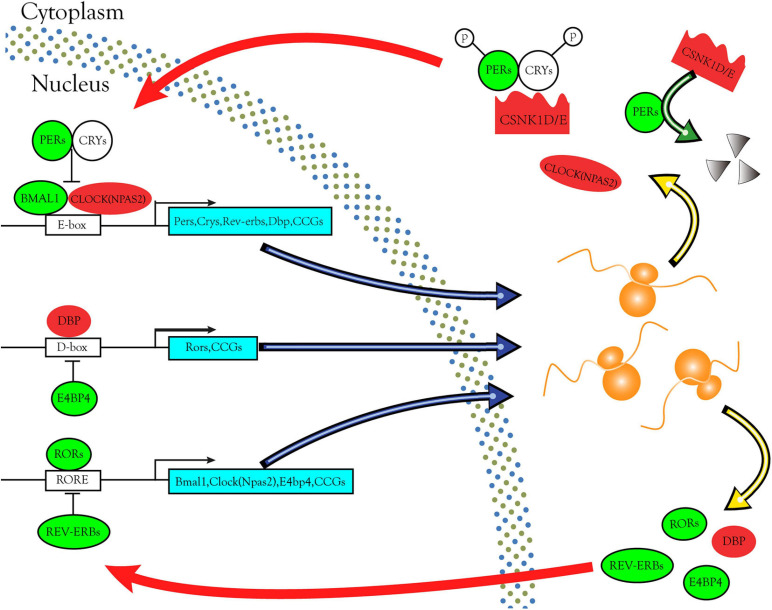
Circadian rhythm pathways enriched in DERGs. The expression levels of DERGs are upregulated and downregulated in LIHC patients, which are indicated in the red and green modules, respectively.

Through bioinformatics analysis and experimental verification, we found that the *CSNK1D*, *CSNK1E*, and *NPAS2* mRNA levels were strongly upregulated in LIHC samples. Moreover, *CSNK1D*, *CSNK1E*, and *NPAS2* are also three key genes in the negative feedback loop of circadian rhythm. In the core loop, two E-box specific transcription factors, CLOCK [Circadian Locomotor Output Cycles Kaput, replaced by NPAS2 (Neuronal PAS domain protein 2)] and BMAL1 (Brain and muscle Arnt-like protein-1) heterodimerize during the day and activate transcription from genes containing E-boxes, including the *Pers*, *Crys*, and clock-controlled genes (CCGs). In the cytoplasm, PERs and CRYs, phosphorylated by CSNK1D/E and from a complex. Subsequently they enter the nucleus where they inhibit CLOCK/BMAL1-mediated transcription in the evening. PERs phosphorylated by CSNK1D/E are degraded *via* the proteasome pathway and restart a new cycle of transcription. Secondary loops include additional pairs of antagonizing transcription factors such as DBP and E4BP4, REV-ERBs, and RORs. DBP and E4BP4 regulate the expression of Rors and other CCGs through D-boxes. REV-ERBs and RORs control the expression of *Bmal1*, *Clock* (*NPAS2*) and other CCGs *via* ROR elements (ROREs) (Figure 9). These interlinked positive and negative transcription-translation feedback loops confer daily rhythmicity for homeostasis maintenance (Lowrey and Takahashi, 2011; Shostak, 2017; Shafi and Knudsen, 2019). Abnormal expression of circadian genes in transcription-translation feedback loops impacts several BPs, such as the cell cycle, apoptosis, DNA damage repair and metabolic regulation, thereby affecting tumor development and progression. However, the molecular mechanism of the occurrence and development of LIHC caused by these three rhythm genes remains to be further studied.

Many anticancer drugs are significantly affected by the time of administration (Smolensky and Peppas, 2007; Lévi et al., 2010; Ye et al., 2018), and their biological behavior, such as absorption, distribution, metabolism, and elimination, follow circadian rhythms. A recent study found that once the drugs are inside the tumor cells, their functions are regulated by the circadian rhythm of the tumor cells, with the result that these anticancer drugs show different anticancer activities and adverse reactions with different times of administration (Lévi et al., 2008; Dulong et al., 2015). Therefore, the identification of these DERGs contributes to be used as diagnostic and prognostic biomarkers and molecular targets for chronotherapy in LIHC patients, which has broad application prospects.

## Data Availability Statement

The original contributions presented in the study are included in the article/Supplementary Material, further inquiries can be directed to the corresponding author/s.

## Author Contributions

HL performed most of the bioinformatics analyses and experiments. HL, SH, and ZF created the figures and tables. HL and XW wrote the manuscript. YG designed, performed, and supervised the study. SL provided suggestions and guidance of this study, and revised the manuscript. All authors contributed to the article and approved the submitted version.

## Conflict of Interest

The authors declare that the research was conducted in the absence of any commercial or financial relationships that could be construed as a potential conflict of interest.

## Footnotes

1 https://portal.gdc.cancer.gov/repository

2 https://www.string-db.org

## Supplementary Material

Supplementary Figure 1 Relationships between DERG expression levels and survival rate in hepatic carcinoma. **(A)** *PER1*, **(B)** *PER2*, **(C)** *PER3*, **(D)** *ARNTL*, **(E)** *NR1D1*, **(F)** *PRKAG2*, and **(G)** *RORA*.

Supplementary Figure 2 Kaplan–Meier survival curves of three hub genes in hepatic carcinoma patients. **(A)** *CSNK1D*, **(B)** *CSNK1K*, and **(C)** *NPAS2*.

Supplementary Table 1 The 563 differentially expressed rhythm genes.

Supplementary Table 2 Information on the 377 clinical samples of LIHC.

Supplementary Table 3 Gene (*CSNK1D*, *CSNK1E*, and *NPAS2*) expression profiles of 50 pairs of normal and tumor samples from the same patients.

Supplementary Table 4 Univariate and multivariate analyses of prognostic factors (CSNK1D).

Supplementary Table 5 Univariate and multivariate analyses of prognostic factors (CSNK1E).

Supplementary Table 6 Univariate and multivariate analyses of prognostic factors (NPAS2).

Supplementary Table 7 Raw data from the 235 patient samples of Cox analysis.
