# Supplementary material for: Bioinformatics Analysis of Differentially Expressed Rhythm Genes in Liver Hepatocellular Carcinoma
Source: Front Genet. 2021 Jun 3;12:680528. doi: 10.3389/fgene.2021.680528 (PMC8211427; doi:10.3389/fgene.2021.680528)
Supplement: Supplementary Figure 1 — Relationships between DERG expression levels and survival rate in hepatic carcinoma. (A) PER1, (B) PER2, (C) PER3, (D) ARNTL, (E) NR1D1, (F) PRKAG2, and (G) RORA. [file Data_Sheet_1.docx]

Supplementary Material

# Supplementary Figures and Tables

For more information on Supplementary Material and for details on the different file types accepted, please see [here](http://home.frontiersin.org/about/author-guidelines#SupplementaryMaterial). Figures, tables, and images will be published under a Creative Commons CC-BY licence and permission must be obtained for use of copyrighted material from other sources (including re-published/adapted/modified/partial figures and images from the internet). It is the responsibility of the authors to acquire the licenses, to follow any citation instructions requested by third-party rights holders, and cover any supplementary charges.

## Supplementary Figures

##
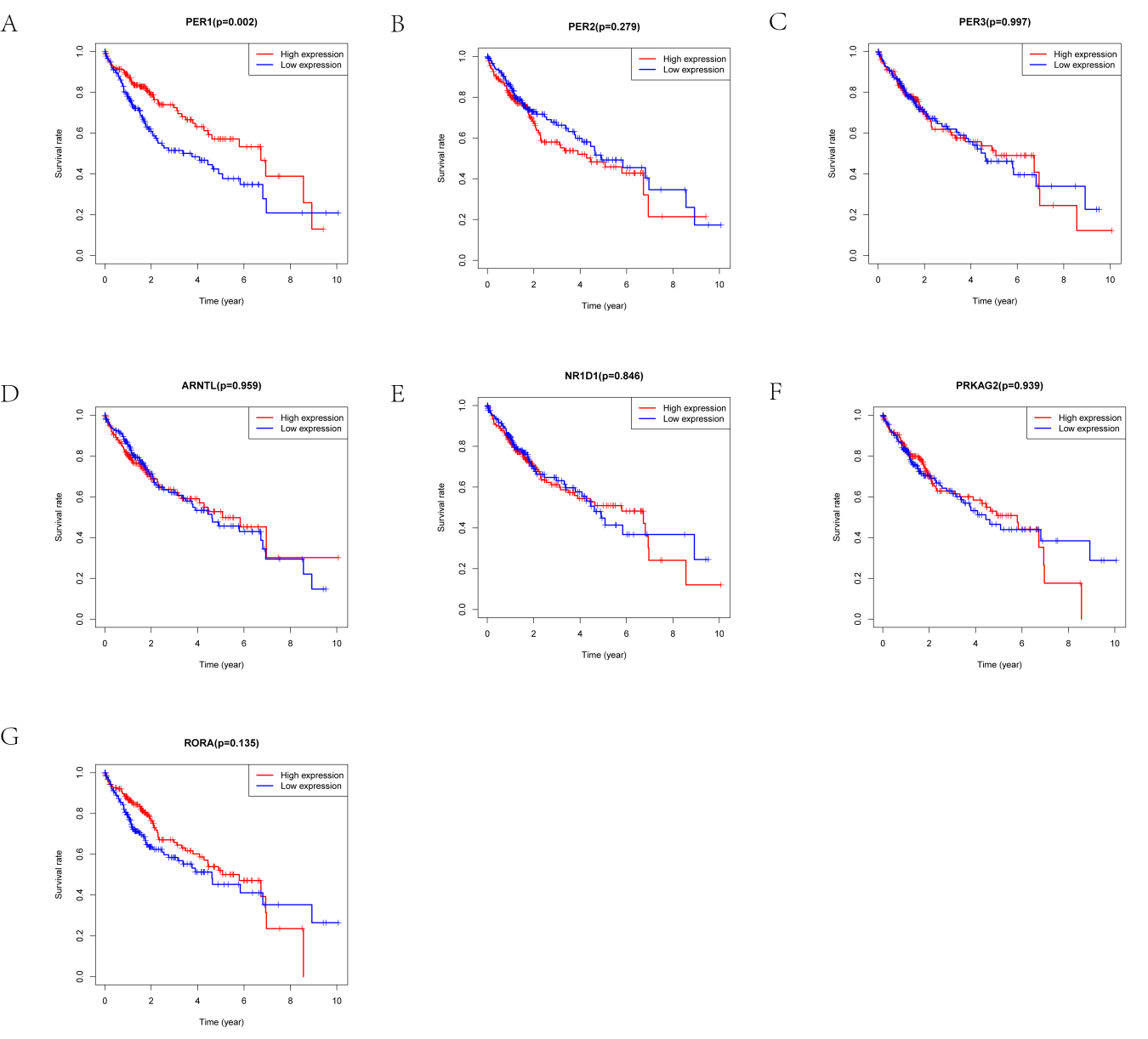


**Supplementary Figure 1.** Relationship between RDEG expression levels and survival rate in hepatic carcinoma. (A) *PER1*, (B) *PER2*, (C) *PER3*, (D) *ARNTL*, (E) *NR1D1*, (F) *PRKAG2*, and (G) *RORA*.


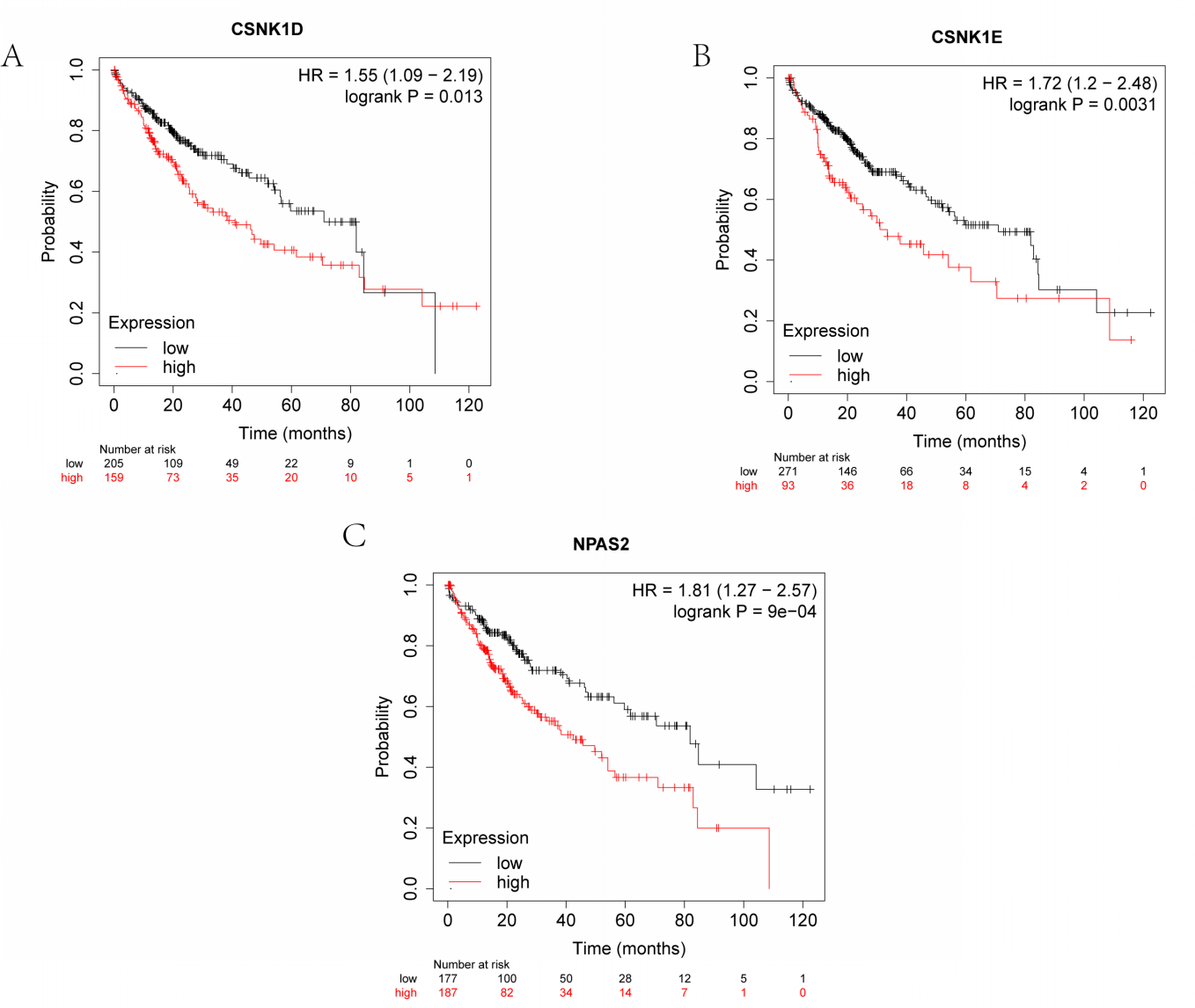


**Supplementary Figure 2.** Kaplan–Meier survival curves of three hub genes in hepatic carcinoma patients. (A) CSNK1D, (B) CSNK1K and (C) NPAS2 expression.

## Supplementary Tables

**Supplementary Table 1.** The 563 Differentially Expressed Rhythm Genes

| gene | logFC | logCPM | PValue | FDR |
| --- | --- | --- | --- | --- |
| ABCA1 | -0.946185241 | 4.420713998 | 2.39E-16 | 4.43E-15 |
| ABCA7 | 0.845218664 | 3.12887832 | 0.000774974 | 0.001942205 |
| ABCD1 | 0.955458727 | 4.621327661 | 1.29E-09 | 9.51E-09 |
| ABCG1 | 0.541143795 | 3.585563019 | 0.020639513 | 0.037209854 |
| ABHD2 | -0.963872671 | 5.990254718 | 1.46E-13 | 1.9E-12 |
| ABHD3 | 0.339291712 | 4.722488561 | 0.017503966 | 0.032073817 |
| ABHD4 | 0.794269585 | 4.933845562 | 3.61E-09 | 2.46E-08 |
| ABHD5 | -0.502359432 | 3.830994407 | 0.0000473 | 0.000152629 |
| ACADSB | -1.743721138 | 6.810359087 | 4.3E-37 | 6.76E-35 |
| ACOX1 | -0.94540206 | 6.314722833 | 2.49E-15 | 4.11E-14 |
| ACSL1 | -1.809239401 | 8.076124695 | 1.07E-29 | 8.28E-28 |
| ACYP1 | 1.042688193 | 3.11732887 | 0.0000897 | 0.000274025 |
| ADIPOR1 | 0.322315649 | 6.845450937 | 0.0000544 | 0.000173485 |
| ADIPOR2 | -0.593849193 | 5.946123716 | 6.05E-09 | 4.03E-08 |
| ADM | -1.300761659 | 4.640039116 | 4.46E-15 | 7.17E-14 |
| AGMAT | -0.984663784 | 6.270567921 | 5.34E-15 | 8.46E-14 |
| AGTRAP | 0.534988346 | 4.750642012 | 0.0000942 | 0.00028623 |
| AK3 | -0.956682069 | 6.227451418 | 1.3E-19 | 3.68E-18 |
| ALKBH2 | 0.700411272 | 4.932822418 | 0.000000818 | 0.00000368 |
| ALPL | -1.552869345 | 5.714174942 | 1.89E-13 | 2.43E-12 |
| ANAPC4 | 0.5276531 | 3.375038974 | 0.013776345 | 0.025940053 |
| ANGEL2 | 0.44214794 | 3.574325556 | 0.018585271 | 0.033851457 |
| ANKRD13D | 0.76666755 | 3.409604024 | 0.000255346 | 0.000710166 |
| ANKS6 | 1.233899328 | 3.077376252 | 0.0000141 | 0.0000506 |
| AOAH | -0.717991895 | 3.211329947 | 0.00026462 | 0.000734477 |
| AOC3 | -0.677862127 | 3.9127312 | 0.00000165 | 0.00000701 |
| AP1G1 | -0.422061676 | 4.267143427 | 0.0000261 | 0.0000884 |
| AP3M2 | 0.86004351 | 3.026797753 | 0.002638165 | 0.005897522 |
| APCDD1 | 1.560684158 | 3.528874828 | 0.001634707 | 0.003829427 |
| APOBEC3B | 1.628016999 | 3.357407683 | 8.23E-08 | 0.000000445 |
| APOL1 | -1.010431889 | 7.411681753 | 1.03E-14 | 1.57E-13 |
| AQP3 | -1.524795487 | 5.971513755 | 5.53E-27 | 3.35E-25 |
| AQP9 | -0.68332751 | 8.58528725 | 0.00762539 | 0.015253236 |
| ARG1 | -1.07789187 | 8.992970416 | 0.00000035 | 0.00000169 |
| ARID3A | 2.255801366 | 3.498117829 | 5.5E-10 | 4.26E-09 |
| ARID5B | -0.336298131 | 3.322933536 | 0.028794247 | 0.04990467 |
| ARMC1 | 0.542896683 | 4.913616836 | 0.000000257 | 0.00000127 |
| ARNTL | -0.377338647 | 3.285547436 | 0.021690169 | 0.038883863 |
| ARV1 | 0.454651752 | 4.947852042 | 0.0000214 | 0.0000739 |
| ASB13 | -0.576965364 | 5.613719769 | 0.00000561 | 0.0000217 |
| ASL | -0.594762177 | 7.250039066 | 0.000113757 | 0.000340842 |
| ASNS | 1.537296965 | 3.434044303 | 0.000000937 | 0.00000416 |
| ATG2A | -0.782222333 | 4.446916101 | 2.09E-16 | 3.93E-15 |
| ATIC | 0.612887329 | 5.618731936 | 1.14E-08 | 7.23E-08 |
| ATP1B1 | 0.740786612 | 8.084318895 | 0.00000575 | 0.0000222 |
| ATP6V0B | 0.433454919 | 6.618134569 | 0.0000841 | 0.000258236 |
| ATP8B2 | 0.616358504 | 3.296157012 | 0.007516529 | 0.015064599 |
| AVIL | 1.703718941 | 3.094707115 | 0.0000412 | 0.000134352 |
| AXIN2 | 1.862508782 | 3.391210551 | 0.00000925 | 0.0000344 |
| B4GALT5 | -0.534188446 | 4.922074359 | 0.00000465 | 0.0000182 |
| BCKDHB | -1.502069345 | 4.675916757 | 3.39E-36 | 4.95E-34 |
| BCL3 | -0.850905029 | 5.872178977 | 5.33E-11 | 4.8E-10 |
| BCL6 | -0.395517772 | 4.55209904 | 0.002319086 | 0.005256143 |
| BEX2 | 2.430003564 | 4.354197398 | 1.33E-08 | 8.37E-08 |
| BIRC3 | 0.733960333 | 4.71179324 | 0.003908702 | 0.008400529 |
| BRIX1 | 0.456652721 | 3.918299719 | 0.002826508 | 0.006282366 |
| BRWD1 | -0.35600926 | 3.322042934 | 0.024163593 | 0.042756269 |
| BSG | 1.023203757 | 8.177699127 | 3.79E-15 | 6.13E-14 |
| BTNL8 | 2.667506638 | 3.096579761 | 0.00000085 | 0.00000381 |
| BZW2 | 0.690254813 | 4.622811726 | 0.000000557 | 0.00000258 |
| C12orf66 | -0.52725663 | 3.096571813 | 0.003344777 | 0.007292005 |
| C1QA | -0.869298962 | 8.014776559 | 0.00000137 | 0.0000059 |
| C1QB | -1.049718776 | 7.715535266 | 7.81E-09 | 5.11E-08 |
| C1RL | -1.643283875 | 6.342649217 | 4.2E-56 | 1.86E-53 |
| C20orf27 | 0.820856308 | 5.133168301 | 3.7E-09 | 2.52E-08 |
| C3orf18 | 0.687019952 | 3.325031991 | 0.001441781 | 0.003414196 |
| C3orf62 | 0.603932397 | 3.145012302 | 0.01542432 | 0.028695012 |
| C4BPA | -0.831085468 | 10.3067967 | 0.0000298 | 0.0000998 |
| C5AR1 | -1.078281429 | 3.64551632 | 2.58E-12 | 2.83E-11 |
| CABYR | 3.185579882 | 3.297067847 | 3.35E-10 | 2.67E-09 |
| CALCOCO2 | -0.270116354 | 5.12347191 | 0.000946478 | 0.00232832 |
| CANT1 | 0.702554546 | 4.897907562 | 5.82E-11 | 5.22E-10 |
| CAPRIN1 | 0.35311594 | 5.331288273 | 0.0000667 | 0.000208855 |
| CASK | 0.548578702 | 3.715628272 | 0.001280418 | 0.003072509 |
| CCDC88A | 0.650010477 | 3.131800945 | 0.011810683 | 0.022626513 |
| CD200 | 1.526966671 | 2.963436674 | 0.00000116 | 0.00000506 |
| CD248 | 1.108946814 | 4.157444025 | 0.000000491 | 0.0000023 |
| CD46 | 0.472666049 | 5.970265465 | 0.0000182 | 0.0000638 |
| CD83 | -0.838003896 | 3.543890759 | 0.00000002 | 0.000000122 |
| CDA | -1.344868976 | 5.851107558 | 1.44E-11 | 1.4E-10 |
| CDC42SE1 | 0.468162505 | 5.061004231 | 0.0000246 | 0.0000839 |
| CDCA7L | 0.820427007 | 3.184937976 | 0.003474838 | 0.007551721 |
| CEACAM1 | -0.306169616 | 5.297109095 | 0.01952171 | 0.035395035 |
| CENPH | 1.711232419 | 3.302245434 | 3.71E-11 | 3.41E-10 |
| CEP68 | 0.597283421 | 3.167967053 | 0.014090523 | 0.026459416 |
| CFLAR | -0.275830389 | 3.78139305 | 0.024787421 | 0.043723039 |
| CHCHD7 | 0.449703084 | 3.929295033 | 0.002784701 | 0.006197208 |
| CHIC2 | -0.951100408 | 4.361787346 | 5.79E-27 | 3.49E-25 |
| CHMP1A | 0.216228924 | 5.772519469 | 0.015247284 | 0.028386922 |
| CHPF2 | 0.339869921 | 4.933154868 | 0.000975057 | 0.002392937 |
| CHST11 | 0.647824247 | 3.247396162 | 0.019238925 | 0.034928212 |
| CHST7 | -1.028640788 | 3.617724295 | 4.36E-10 | 3.42E-09 |
| CLYBL | -0.95015625 | 4.127992529 | 1.69E-13 | 2.18E-12 |
| CORO1C | -0.314231809 | 5.025962513 | 0.000592168 | 0.001524367 |
| COTL1 | -0.544070637 | 4.412672014 | 0.000857776 | 0.002132506 |
| CPD | 0.776407679 | 5.308986055 | 0.000000248 | 0.00000123 |
| CPNE7 | 2.001493535 | 2.943580819 | 0.00000417 | 0.0000165 |
| CPPED1 | 0.301519487 | 5.022628961 | 0.02462836 | 0.04346658 |
| CRAT | -0.680258822 | 6.750029919 | 4.39E-13 | 5.3E-12 |
| CRISPLD2 | -1.007842429 | 3.491446464 | 0.00000143 | 0.00000616 |
| CSAD | -0.90388947 | 4.550298347 | 5.77E-10 | 4.46E-09 |
| CSNK1D | 0.473144581 | 4.861836584 | 0.00000127 | 0.00000552 |
| CSNK1E | 0.696786502 | 4.49595107 | 8.27E-08 | 0.000000447 |
| CSRNP1 | -2.228210781 | 5.210957232 | 3.34E-80 | 3.78E-77 |
| CST7 | -0.593160079 | 4.043713421 | 0.003660639 | 0.00791948 |
| CTBS | -1.030188008 | 3.852951554 | 4.34E-20 | 1.3E-18 |
| CTSS | -0.417297682 | 6.045950955 | 0.007388423 | 0.014834185 |
| CUEDC1 | 1.036353369 | 3.754640781 | 2.12E-08 | 0.000000128 |
| CXCL2 | -1.55699764 | 6.277266733 | 5.3E-15 | 8.4E-14 |
| CYBRD1 | -0.655173236 | 4.081954107 | 0.000187685 | 0.000537766 |
| CYCS | 0.376493863 | 6.064721791 | 0.001517133 | 0.003577623 |
| CYHR1 | 0.859487114 | 4.465520296 | 3.25E-09 | 2.23E-08 |
| CYP27A1 | -0.855434725 | 9.026011789 | 5.43E-08 | 0.000000305 |
| CYP2J2 | -1.120307227 | 6.21875387 | 9.28E-14 | 1.24E-12 |
| CYP4F2 | -1.596397926 | 6.670677122 | 6.36E-16 | 1.12E-14 |
| CYP4V2 | -1.427664877 | 4.954804579 | 8.54E-28 | 5.73E-26 |
| DBN1 | 1.683399088 | 4.185819593 | 3.47E-10 | 2.76E-09 |
| Dbp | 0.538398779 | 3.719365037 | 0.002424106 | 0.005464249 |
| DCAF4 | 0.556673069 | 3.15012328 | 0.027579906 | 0.048061644 |
| DDR1 | 1.184210153 | 4.022795701 | 0.0000654 | 0.000204913 |
| DDX24 | -0.225825893 | 4.966307968 | 0.007163093 | 0.014428434 |
| DDX42 | 0.231971939 | 4.741490723 | 0.025357322 | 0.044620551 |
| DENND4A | -0.653087446 | 3.58353927 | 0.000001 | 0.00000443 |
| DENND5B | -0.482849598 | 3.4219785 | 0.000970669 | 0.002384336 |
| DGAT2 | -1.046530923 | 5.954469611 | 1.17E-10 | 9.9E-10 |
| DHODH | -1.775163455 | 4.804804736 | 1.6E-32 | 1.7E-30 |
| DHX34 | 1.120392205 | 3.928926089 | 5.19E-12 | 5.38E-11 |
| DNAJC10 | 0.599212433 | 3.396722902 | 0.005029874 | 0.010524174 |
| DNAJC16 | -0.692286771 | 3.747328796 | 0.000000025 | 0.00000015 |
| DNM1L | 0.473125612 | 4.064044749 | 0.001011391 | 0.002473308 |
| Dnmt1 | 1.173714626 | 3.894842477 | 5E-10 | 3.89E-09 |
| DOK4 | 0.387399834 | 4.802905094 | 0.006545226 | 0.013330919 |
| DUSP1 | -1.656410045 | 8.388302235 | 1.56E-27 | 1.01E-25 |
| DUSP12 | 0.932339629 | 4.259154151 | 4.17E-12 | 4.41E-11 |
| DVL3 | 0.698785087 | 4.905482138 | 1.59E-10 | 1.31E-09 |
| DYRK2 | 0.646035131 | 3.244907256 | 0.008832902 | 0.017438312 |
| ECE1 | -0.678649445 | 5.481357335 | 5.25E-14 | 7.3E-13 |
| ECHDC2 | -0.744589594 | 5.479259165 | 0.000000027 | 0.000000161 |
| ECHDC3 | -0.841984474 | 6.724947219 | 0.000000408 | 0.00000195 |
| EHMT2 | 1.316978761 | 4.496185615 | 3.03E-20 | 9.31E-19 |
| EID1 | -0.258040522 | 6.707831679 | 0.005659672 | 0.01171754 |
| EIF3E | 0.642106018 | 6.690045588 | 0.00000224 | 0.00000931 |
| EIF3M | 0.216375575 | 5.2094419 | 0.011986924 | 0.022904081 |
| EIF4B | -0.188286283 | 6.595753589 | 0.02717543 | 0.047423341 |
| ELAVL1 | 0.252814878 | 4.614569773 | 0.017748167 | 0.032488163 |
| ELL | -0.291835117 | 3.745107494 | 0.023286259 | 0.041368922 |
| ELP2 | -0.396071436 | 4.223418984 | 0.000165445 | 0.000479686 |
| EMP1 | -0.745826038 | 3.829857326 | 0.0000846 | 0.000259636 |
| EPHX2 | -1.51629107 | 6.353733893 | 5.77E-26 | 3.06E-24 |
| EPS15L1 | 0.675837354 | 3.462727512 | 0.000862647 | 0.002142899 |
| ERGIC1 | -0.466298657 | 5.586080769 | 0.00000071 | 0.00000322 |
| EXOSC8 | 0.360783877 | 3.840808718 | 0.016867648 | 0.031068297 |
| EXOSC9 | 0.525055245 | 3.844761586 | 0.000637162 | 0.001629381 |
| EXTL3 | 0.579716945 | 3.348819474 | 0.009350291 | 0.018322852 |
| F12 | -0.965652822 | 8.987393699 | 8.85E-08 | 0.000000475 |
| FAAH2 | -0.737509818 | 3.993940646 | 0.00000304 | 0.0000123 |
| FABP5 | 1.57647172 | 3.687971325 | 2.78E-09 | 1.93E-08 |
| FAM102A | -0.560909455 | 4.969677456 | 0.0000314 | 0.000104536 |
| FAM120A | -0.175408827 | 5.732885983 | 0.010625864 | 0.020588112 |
| FAM162A | -0.334470208 | 5.961645516 | 0.004888034 | 0.010253313 |
| FAM53B | 0.892048676 | 3.45738625 | 0.0000097 | 0.0000359 |
| FANCF | 0.481558282 | 3.660990802 | 0.00839665 | 0.016653826 |
| FBLN5 | -0.889682403 | 4.253123692 | 0.0000102 | 0.0000375 |
| FBXO21 | -0.625784997 | 3.956873761 | 6.99E-08 | 0.000000383 |
| FBXO28 | -0.434840046 | 4.041058961 | 0.0000548 | 0.00017448 |
| FCGBP | 1.272288643 | 2.940709021 | 0.002183013 | 0.004968595 |
| FGR | -0.791965019 | 3.166222163 | 0.0000118 | 0.0000427 |
| FHIT | 0.815187392 | 3.405588414 | 0.000185314 | 0.000531461 |
| FITM2 | 0.497468641 | 3.817657462 | 0.002835545 | 0.006299069 |
| FKBP1A | 0.532603041 | 6.95197737 | 1.79E-08 | 0.000000111 |
| FLOT1 | 0.449606079 | 7.016915008 | 0.00000295 | 0.000012 |
| FOS | -3.06081895 | 6.690326465 | 3.6E-58 | 1.65E-55 |
| FOXJ1 | 2.395389191 | 3.014938037 | 0.0000186 | 0.0000651 |
| FOXK1 | 1.143281545 | 3.16739181 | 0.0000176 | 0.0000619 |
| FOXO1 | -1.686192211 | 4.270992162 | 6.78E-35 | 8.33E-33 |
| G6PD | 2.538434369 | 5.05051558 | 1.45E-18 | 3.61E-17 |
| GAB2 | 0.649111242 | 3.479764489 | 0.000861182 | 0.002140115 |
| GABARAPL1 | -1.063543346 | 6.051239793 | 2.19E-13 | 2.77E-12 |
| GADD45B | -1.936933677 | 7.593360533 | 4.18E-36 | 6.04E-34 |
| GATM | -1.191282639 | 8.646967119 | 3.86E-14 | 5.5E-13 |
| GCSH | -0.816919958 | 4.205907774 | 3.55E-13 | 4.34E-12 |
| GGA2 | 0.432091736 | 4.466250226 | 0.000344699 | 0.000934806 |
| GK | -0.657931108 | 4.427970128 | 0.0000718 | 0.000223269 |
| GLT1D1 | -0.985572879 | 4.507657356 | 3.14E-08 | 0.000000185 |
| GNAI2 | -0.27247832 | 6.203973273 | 0.0000972 | 0.000294445 |
| GNMT | -1.493074038 | 7.895465259 | 6.68E-09 | 4.42E-08 |
| GNS | 0.374598869 | 6.169650986 | 0.000115498 | 0.000345687 |
| GON4L | 0.704520737 | 3.282202232 | 0.002789141 | 0.006204863 |
| GPATCH4 | 0.826094691 | 4.62934219 | 1.03E-10 | 8.88E-10 |
| GPX7 | 0.83299277 | 3.917239526 | 0.000580796 | 0.001496955 |
| Grem2 | -0.995092989 | 4.170215796 | 0.000889128 | 0.002200756 |
| GRPEL1 | -0.691728182 | 5.171222305 | 8.06E-16 | 1.41E-14 |
| HAAO | -1.368537926 | 7.245166027 | 4.39E-19 | 1.18E-17 |
| HABP4 | 0.49475612 | 3.835190013 | 0.008157184 | 0.01620994 |
| HACL1 | -0.301799984 | 5.162014613 | 0.008606893 | 0.017035447 |
| HAL | -0.732518657 | 6.16868938 | 0.004354966 | 0.009255514 |
| HBP1 | -0.349797062 | 4.204269427 | 0.002827446 | 0.006283325 |
| HCK | -0.772633249 | 3.66662884 | 0.00000549 | 0.0000212 |
| HHAT | 0.76211247 | 3.106627271 | 0.004263474 | 0.00908283 |
| HK3 | -1.264457719 | 3.020828755 | 7.4E-11 | 6.5E-10 |
| HKDC1 | 1.600175887 | 4.791518051 | 2.38E-09 | 1.67E-08 |
| HLA-DOA | -0.553499381 | 3.671547581 | 0.009028778 | 0.017765706 |
| HLCS | -0.294501947 | 3.616756258 | 0.025852129 | 0.045337063 |
| HNRNPA1 | 0.601742793 | 7.204920918 | 2.43E-11 | 2.28E-10 |
| HNRNPA3 | 0.350803314 | 6.147492455 | 0.00000247 | 0.0000102 |
| HNRNPU | 0.343127823 | 6.335130452 | 0.000000287 | 0.0000014 |
| HOOK1 | -0.815352991 | 4.144128957 | 9.24E-14 | 1.23E-12 |
| HP | -2.030555311 | 12.28000315 | 4.27E-21 | 1.43E-19 |
| HPRT1 | -0.284779985 | 5.678892548 | 0.018322589 | 0.033435696 |
| HPS4 | 0.552499704 | 3.397788833 | 0.010629655 | 0.020588112 |
| HSD17B8 | -0.455117384 | 6.545908814 | 0.000665095 | 0.001693144 |
| HSF2 | 0.609702414 | 3.487265216 | 0.00244471 | 0.0055037 |
| HSPA14 | 0.505086476 | 3.632772086 | 0.005138758 | 0.010728497 |
| HSPA1A | 0.774659405 | 7.226233325 | 0.0000187 | 0.0000654 |
| HSPA1B | 0.667758168 | 6.272525057 | 0.003182448 | 0.006971183 |
| HSPA6 | 1.210825767 | 3.906098659 | 0.001304104 | 0.003122107 |
| HTATSF1 | 0.353933154 | 5.554162582 | 0.000211887 | 0.000600186 |
| IBTK | -0.273049046 | 4.665149123 | 0.005924312 | 0.012208448 |
| IDH1 | -0.406756744 | 7.41506598 | 0.0000526 | 0.000168183 |
| IDS | -0.402243214 | 3.947957858 | 0.000992377 | 0.002430159 |
| IFIT2 | -0.799225366 | 4.445294335 | 0.0000113 | 0.000041 |
| IFIT3 | -0.744790461 | 5.143924357 | 0.00000633 | 0.0000242 |
| IGHD | -1.352514589 | 3.137705978 | 0.0000224 | 0.0000771 |
| IKBIP | 0.602003412 | 3.726005099 | 0.000386375 | 0.001036509 |
| IL13RA1 | -0.58152976 | 6.567047148 | 0.000000016 | 9.93E-08 |
| IL1R1 | -0.795867018 | 5.18523274 | 1.34E-10 | 1.12E-09 |
| IL1R2 | 0.710689878 | 3.700606258 | 0.001207174 | 0.002917044 |
| IL1RAP | -1.928271076 | 3.784456619 | 7.75E-44 | 1.93E-41 |
| IL1RN | -2.131456864 | 5.864058722 | 7.59E-39 | 1.37E-36 |
| IL6ST | -0.991140584 | 6.006533782 | 3.42E-26 | 1.86E-24 |
| IMPDH1 | 0.907826219 | 3.864741978 | 0.000181075 | 0.000520749 |
| INHBB | -0.513883449 | 5.319014526 | 0.002332976 | 0.005282721 |
| IP6K1 | 0.877737588 | 4.607987882 | 1.35E-14 | 2.01E-13 |
| IQCB1 | 0.651351998 | 3.59950151 | 0.000563937 | 0.001456826 |
| IRF1 | -0.489574433 | 4.563863389 | 0.000358358 | 0.000968041 |
| IRF2 | -0.419783018 | 4.763223219 | 0.000000513 | 0.0000024 |
| IRF7 | -0.360683301 | 5.320443746 | 0.01633721 | 0.030197224 |
| IRS2 | -0.525394623 | 5.108602039 | 0.000471521 | 0.001238702 |
| ITCH | -0.691856969 | 4.741890836 | 3.85E-14 | 5.49E-13 |
| ITGA5 | 0.586299201 | 5.308538756 | 0.0000343 | 0.000113487 |
| ITGA6 | 1.382620709 | 4.851113561 | 1.53E-15 | 2.6E-14 |
| ITPK1 | 0.36645278 | 5.427348955 | 0.001554907 | 0.003663221 |
| JDP2 | -1.117731639 | 3.272456687 | 1.06E-13 | 1.4E-12 |
| JUNB | -1.52863203 | 7.359255452 | 5.95E-31 | 5.2E-29 |
| KANK1 | -0.447198066 | 4.6303722 | 0.000301673 | 0.000826787 |
| KCNE3 | 0.698059388 | 3.228581329 | 0.004825438 | 0.010137419 |
| KDSR | -0.921153031 | 4.446876082 | 3.84E-26 | 2.07E-24 |
| KIF11 | 1.98595994 | 3.230398053 | 6.23E-11 | 5.54E-10 |
| KLF11 | -1.187225588 | 4.527340122 | 8.8E-27 | 5.11E-25 |
| KLF12 | -0.597073575 | 3.414867488 | 0.0000753 | 0.000233102 |
| Klf9 | -0.966439036 | 5.884742126 | 8.5E-11 | 7.4E-10 |
| KLHDC8B | 0.69407562 | 4.833137738 | 0.00000785 | 0.0000295 |
| KPNA1 | -0.304795429 | 4.050706931 | 0.007327016 | 0.014718033 |
| KRT23 | 1.653641203 | 5.17543134 | 0.000405766 | 0.001082531 |
| LANCL1 | 0.4346052 | 4.637566305 | 0.000426306 | 0.001130928 |
| LARP4 | -0.565405477 | 4.784792812 | 8.31E-11 | 7.24E-10 |
| LATS2 | -0.682625698 | 3.633790331 | 0.000000157 | 0.000000809 |
| LCN2 | 4.671958444 | 8.394868884 | 1.78E-17 | 3.85E-16 |
| LDLR | -1.490645671 | 5.360175736 | 4.18E-31 | 3.79E-29 |
| LEF1 | 2.267465223 | 3.048661702 | 1.63E-09 | 1.18E-08 |
| LEPR | -0.783201494 | 5.425338606 | 0.000529643 | 0.001375964 |
| LIMK2 | 0.964562559 | 4.13632787 | 0.000000272 | 0.00000133 |
| LMNB1 | 1.24950572 | 4.387323918 | 3.62E-10 | 2.87E-09 |
| LMO7 | -0.500271372 | 3.928267605 | 0.0000422 | 0.000137292 |
| LPAR2 | 0.885268467 | 3.205533418 | 0.006489564 | 0.013234916 |
| LPCAT1 | 1.368448103 | 4.367993533 | 1.68E-09 | 1.21E-08 |
| LRG1 | -1.659826248 | 9.107142638 | 8.18E-22 | 2.92E-20 |
| LRIG1 | -0.391000818 | 4.331117671 | 0.001632064 | 0.003824679 |
| LRP10 | 0.32261355 | 5.434123906 | 0.006038515 | 0.012421117 |
| LUC7L3 | 0.5431974 | 4.483771964 | 0.00000234 | 0.00000972 |
| LYNX1 | 0.564402723 | 4.73330208 | 0.009520754 | 0.018630431 |
| MAGED1 | 0.381766646 | 6.218575557 | 0.011090014 | 0.02138112 |
| MAGOH | 0.266869757 | 5.556786728 | 0.002131453 | 0.004863635 |
| MAN1A1 | -1.156776489 | 6.466291986 | 1E-22 | 3.98E-21 |
| MAN1C1 | -1.607227091 | 3.503417065 | 1.86E-19 | 5.16E-18 |
| MAP1LC3B | -0.287043955 | 4.793767605 | 0.010473173 | 0.020311708 |
| MAP7 | -0.735380847 | 4.052718359 | 2.92E-11 | 2.71E-10 |
| MAPKAPK2 | 0.564217795 | 6.180275244 | 2.03E-08 | 0.000000124 |
| MAST3 | 0.873658139 | 3.851979185 | 5.39E-08 | 0.000000303 |
| MBOAT7 | 0.580706085 | 4.866044247 | 0.000000364 | 0.00000175 |
| MCL1 | -1.242828453 | 7.693691715 | 1.26E-44 | 3.27E-42 |
| MDC1 | 0.709201003 | 3.857053524 | 0.0000187 | 0.0000653 |
| MED25 | 0.576456588 | 4.1540678 | 0.0000184 | 0.0000644 |
| METTL1 | 0.521979523 | 4.231140581 | 0.000204331 | 0.000580372 |
| METTL3 | 0.675334415 | 3.734379844 | 0.0000872 | 0.000267015 |
| METTL5 | 0.523967857 | 4.636017956 | 0.00000149 | 0.00000639 |
| MIDN | -0.58650003 | 5.281390534 | 0.000000275 | 0.00000135 |
| MORC2 | 0.59633539 | 4.289521462 | 0.00000318 | 0.0000128 |
| MPZL1 | 0.876861431 | 4.814578899 | 4.47E-11 | 4.06E-10 |
| MRTO4 | 0.408279568 | 5.041821306 | 0.000345992 | 0.000938106 |
| MSL1 | 0.355758891 | 4.44461976 | 0.00272094 | 0.006066183 |
| MTA3 | 0.75589983 | 3.26645425 | 0.001595297 | 0.003747001 |
| MTHFR | 0.473213611 | 3.317346258 | 0.025752635 | 0.045207467 |
| MTHFS | -0.663514264 | 5.258748097 | 0.000000619 | 0.00000284 |
| MTR | 0.718053324 | 3.705231314 | 0.000250694 | 0.000698635 |
| MUC20 | 0.793417302 | 3.412670791 | 0.021233163 | 0.03816376 |
| MXD3 | 2.289696001 | 3.295367021 | 3.32E-16 | 6.07E-15 |
| MYBBP1A | 0.326536311 | 4.337041069 | 0.00909757 | 0.017875774 |
| MYC | -1.18066023 | 5.758780282 | 1.15E-11 | 1.14E-10 |
| MYL9 | 1.020964915 | 6.615564956 | 0.000392024 | 0.001050756 |
| MYO1F | -0.778821758 | 3.197498023 | 0.00000287 | 0.0000117 |
| N4BP2 | -0.586676743 | 3.09213149 | 0.000770708 | 0.001933074 |
| NADK | -0.509775194 | 5.204657059 | 9.05E-08 | 0.000000485 |
| NAE1 | 0.355930138 | 4.162062951 | 0.006353388 | 0.012980648 |
| NAMPT | -1.500891925 | 5.799932569 | 9.92E-22 | 3.5E-20 |
| NAT8B | 1.677658969 | 3.982707978 | 0.000000769 | 0.00000347 |
| NDEL1 | -0.856865731 | 3.61244629 | 4.04E-11 | 3.69E-10 |
| NDRG2 | -1.49182537 | 6.237979393 | 1.28E-35 | 1.81E-33 |
| NDUFAF2 | 0.673462266 | 5.223263852 | 0.000000134 | 0.000000698 |
| NET1 | -0.218561488 | 5.105074437 | 0.02170772 | 0.038909709 |
| NFE2L3 | 0.992427536 | 3.286574686 | 0.000254691 | 0.000708501 |
| NFKB2 | 0.648300167 | 5.323043248 | 9.97E-08 | 0.000000531 |
| NOP58 | 0.4344115 | 5.497846134 | 0.00000476 | 0.0000186 |
| NOSIP | 0.402174571 | 4.976352924 | 0.00297711 | 0.006573569 |
| NPAS2 | 0.61615936 | 3.167156731 | 0.014090523 | 0.026459416 |
| NPM1 | 0.737233707 | 7.681403319 | 1.64E-11 | 1.58E-10 |
| NR1D1 | -0.572715579 | 5.243094558 | 0.0000632 | 0.000198864 |
| Nr1d2 | -0.420515537 | 4.285737453 | 0.000139197 | 0.000410191 |
| NR3C2 | -1.250306782 | 3.02150785 | 9.72E-13 | 1.13E-11 |
| NRBF2 | -1.093139615 | 5.197514011 | 2.8E-39 | 5.35E-37 |
| NRCAM | 1.982616696 | 3.08750835 | 0.0000218 | 0.0000752 |
| NSFL1C | 0.324446587 | 5.127907061 | 0.000322458 | 0.000878133 |
| NT5DC1 | -0.779186531 | 4.072689274 | 1.85E-12 | 2.06E-11 |
| NT5E | -0.765589401 | 5.096608818 | 0.00000104 | 0.00000456 |
| NUCB1 | -0.223387822 | 8.261282978 | 0.011464141 | 0.022030594 |
| NUCB2 | 0.482740998 | 4.585136426 | 0.001044181 | 0.002545974 |
| NUCKS1 | 0.277763968 | 6.960842075 | 0.001248996 | 0.003006986 |
| NUDCD1 | 0.840611483 | 3.678180208 | 0.00000258 | 0.0000106 |
| NUP107 | 0.698261155 | 3.576768271 | 0.000283026 | 0.00078068 |
| NUP98 | -0.274490889 | 4.374659004 | 0.003181728 | 0.006970836 |
| OASL | -1.12673462 | 4.617785206 | 6.07E-08 | 0.000000338 |
| OAZ2 | -0.344339795 | 4.816097258 | 0.0000565 | 0.000179523 |
| OCIAD2 | -0.528795455 | 6.019476537 | 0.000204948 | 0.000581857 |
| OCRL | 0.850733355 | 4.184870701 | 7.8E-09 | 0.000000051 |
| OFD1 | 0.60434595 | 3.666968442 | 0.000667493 | 0.001698008 |
| OGG1 | 0.707221888 | 3.520918689 | 0.000245937 | 0.000686766 |
| OPLAH | 0.322034201 | 5.5610627 | 0.023373408 | 0.041511867 |
| ORM1 | -1.841160884 | 13.10959626 | 4.25E-24 | 1.97E-22 |
| ORM2 | -1.578272448 | 11.5334662 | 4.72E-19 | 1.25E-17 |
| OSGIN1 | 0.949453777 | 7.363962045 | 0.0000216 | 0.0000746 |
| OXNAD1 | -0.532397444 | 3.478125739 | 0.000363132 | 0.000979235 |
| PABPC1L | 1.609871762 | 3.982439234 | 7.04E-14 | 9.67E-13 |
| PACSIN2 | 0.459615767 | 5.667547561 | 0.0000696 | 0.000217145 |
| PAIP2B | -1.35157436 | 3.53539213 | 1.64E-15 | 2.77E-14 |
| PAK1 | 0.598015579 | 4.061793862 | 0.000034 | 0.000112486 |
| PAQR8 | 1.150978711 | 3.076583444 | 0.000540827 | 0.001402671 |
| PCYOX1 | -0.623259563 | 6.060481381 | 3.61E-09 | 2.47E-08 |
| PDCL3 | 0.541097967 | 4.410875486 | 0.00000437 | 0.0000172 |
| PDLIM7 | 1.220632469 | 4.06824368 | 9.74E-11 | 8.4E-10 |
| PDX1 | 1.516601082 | 3.260730702 | 0.00128722 | 0.003087042 |
| PEBP1 | -0.70570949 | 10.9254662 | 2.46E-10 | 1.99E-09 |
| Per1 | -0.873322032 | 4.895354778 | 1.87E-11 | 1.78E-10 |
| Per2 | -0.617732088 | 3.483618454 | 0.0000166 | 0.0000587 |
| PER3 | -0.549112829 | 3.531058903 | 0.000547025 | 0.001416675 |
| PFKFB3 | -0.704722605 | 4.438379747 | 0.001324617 | 0.003165114 |
| PGS1 | 0.697585396 | 3.553747766 | 0.000293706 | 0.000806733 |
| PHC2 | -0.40156532 | 5.881028773 | 0.0000101 | 0.0000373 |
| PHF10 | -0.280284034 | 4.50057663 | 0.00266158 | 0.005944514 |
| PHF14 | 0.432767027 | 3.815824624 | 0.006585149 | 0.013401243 |
| PHGDH | -1.484229476 | 5.478776483 | 8.49E-16 | 1.48E-14 |
| PHLDB3 | 0.358338212 | 4.066958295 | 0.006521605 | 0.013291531 |
| PICALM | -0.337388193 | 5.733348021 | 0.00000185 | 0.00000782 |
| PIGX | 0.398196283 | 3.667434891 | 0.025365122 | 0.044627953 |
| PIK3IP1 | 0.566379302 | 4.264889222 | 0.000818633 | 0.002044606 |
| PILRA | -0.426133956 | 3.270308359 | 0.015911395 | 0.029517136 |
| PITPNA | -0.525236194 | 4.8439187 | 3.88E-09 | 2.64E-08 |
| PJA1 | 0.419418995 | 3.893218229 | 0.006193437 | 0.012706114 |
| PLAUR | -0.528855787 | 3.27117244 | 0.017400029 | 0.031925762 |
| PLCB2 | -0.655124126 | 2.997724408 | 0.000729583 | 0.001841831 |
| PLEK | -0.712519515 | 3.895124589 | 0.000229862 | 0.000645359 |
| PLOD1 | 0.310521112 | 6.846716433 | 0.007655664 | 0.015301471 |
| PLSCR1 | -0.786730456 | 4.458615564 | 7.88E-09 | 5.15E-08 |
| PMEPA1 | 0.911203468 | 3.895630066 | 0.00478466 | 0.010065376 |
| PNPLA6 | 0.225096784 | 4.486150997 | 0.027613561 | 0.048100043 |
| POLG2 | 0.94855421 | 3.317819876 | 0.000039 | 0.000127974 |
| POLR1E | -0.790508657 | 4.53202934 | 3.82E-15 | 6.17E-14 |
| POLR2K | 0.917242425 | 6.254070235 | 2.11E-16 | 3.96E-15 |
| PPA2 | -0.308453142 | 4.888548961 | 0.003097859 | 0.006807506 |
| PPIL1 | 0.6199001 | 5.295420289 | 3.79E-08 | 0.00000022 |
| PPIL4 | -0.532454081 | 4.085762989 | 0.000000309 | 0.0000015 |
| PPM1F | 0.919015846 | 3.570533356 | 0.00000113 | 0.00000493 |
| PPP1R15A | -0.921637768 | 5.301800963 | 2.88E-12 | 3.12E-11 |
| PPP1R3B | -1.638195614 | 5.575398539 | 1.68E-26 | 9.35E-25 |
| PPP2R5A | 0.45212449 | 6.20511323 | 0.00000813 | 0.0000305 |
| PPP2R5B | 0.388994797 | 3.89388793 | 0.009501217 | 0.018598064 |
| PPP4R1 | 0.320203814 | 4.054222729 | 0.02295437 | 0.040849437 |
| PPT1 | 0.290079659 | 5.575298468 | 0.014181951 | 0.02660696 |
| PRF1 | -0.674425491 | 3.617933724 | 0.000529529 | 0.001375957 |
| PRKAG2 | -0.921913349 | 3.813683652 | 3.34E-13 | 4.1E-12 |
| PRKCA | 0.787636425 | 3.805562054 | 0.00000755 | 0.0000285 |
| PRRG4 | -0.936742755 | 4.151823382 | 1.2E-12 | 1.37E-11 |
| PSAT1 | -1.277931265 | 6.839370354 | 1.95E-16 | 3.7E-15 |
| PSD4 | -0.6283294 | 4.127644929 | 5.06E-09 | 3.39E-08 |
| PSMD8 | 0.208475943 | 6.683943338 | 0.006635503 | 0.013492661 |
| PTMA | 0.41877308 | 8.059350744 | 2.92E-08 | 0.000000173 |
| PTP4A3 | 2.45014957 | 4.82202235 | 2.6E-18 | 6.22E-17 |
| PTPRK | -0.243998291 | 4.578359594 | 0.013602162 | 0.0256354 |
| PYGL | -0.494557331 | 6.358128416 | 0.000178201 | 0.000513435 |
| QKI | -0.482794062 | 3.81374319 | 0.0000375 | 0.000123316 |
| QPCT | 1.20078302 | 3.117588695 | 0.000765052 | 0.001919665 |
| RAB20 | -0.975737832 | 5.740475736 | 8.04E-14 | 1.09E-12 |
| RAB24 | 1.012952255 | 3.953503496 | 1.36E-10 | 1.14E-09 |
| RAB27A | -0.828118183 | 4.158350754 | 1.44E-12 | 1.64E-11 |
| RAB3D | 1.404828684 | 3.160849028 | 0.0000292 | 0.0000977 |
| RAC3 | 0.978848302 | 4.958602092 | 0.000000611 | 0.00000281 |
| RALA | 0.377845477 | 4.779743644 | 0.000170857 | 0.000494453 |
| RALBP1 | 0.248177696 | 4.860117634 | 0.019832506 | 0.03586951 |
| RAMP1 | 1.096079829 | 7.66404853 | 0.0000043 | 0.000017 |
| RARA | 0.328732579 | 5.124841382 | 0.003871755 | 0.008325446 |
| RBM17 | 0.346454681 | 4.539343854 | 0.002499046 | 0.00560874 |
| RBM19 | 0.52598425 | 4.00452868 | 0.000261802 | 0.000727468 |
| RCL1 | -1.461163529 | 4.767891262 | 1.9E-23 | 8.26E-22 |
| REPS1 | -0.759052033 | 3.378653646 | 0.000000186 | 0.000000944 |
| REPS2 | -0.794486035 | 3.100523245 | 0.0000072 | 0.0000273 |
| RFFL | -0.291458637 | 3.984399402 | 0.011976316 | 0.022894379 |
| RGL3 | 0.82222363 | 3.823958403 | 0.0000233 | 0.0000801 |
| RHCG | 1.555808275 | 2.992460656 | 0.000923977 | 0.00227748 |
| RHOB | -1.459601002 | 9.309283801 | 2.04E-27 | 1.3E-25 |
| RHOBTB1 | 1.152143712 | 4.185708172 | 0.000338104 | 0.000917921 |
| RHOG | 0.224484023 | 5.841798872 | 0.021413023 | 0.038453641 |
| RMND5A | -0.43029833 | 5.183436618 | 0.00000834 | 0.0000312 |
| RNF157 | 2.22784564 | 3.390455374 | 1.21E-11 | 1.2E-10 |
| RNF213 | 0.773250695 | 4.380727838 | 2.22E-08 | 0.000000134 |
| RNF24 | 0.935871899 | 2.955670196 | 0.002594908 | 0.005809189 |
| RORA | -0.882424218 | 3.377102244 | 3.99E-08 | 0.00000023 |
| RP9 | 0.467504669 | 3.637146331 | 0.006695849 | 0.013602005 |
| RPA3 | 0.305350568 | 4.528789569 | 0.025051654 | 0.044114951 |
| RPL14 | 0.526200746 | 7.114711058 | 0.0000756 | 0.000234097 |
| RPS23 | 0.403951691 | 7.968422649 | 0.000329396 | 0.000896239 |
| RPS5 | 0.777325607 | 8.384383937 | 2.57E-08 | 0.000000153 |
| RRN3 | -0.617608838 | 4.652078193 | 1.51E-12 | 1.71E-11 |
| RRP15 | 0.561055602 | 3.497722294 | 0.003439312 | 0.007478441 |
| RRS1 | 0.882297804 | 5.500459562 | 1.61E-08 | 9.98E-08 |
| RSAD2 | -1.598423432 | 3.135001268 | 9E-13 | 1.05E-11 |
| RTN2 | 1.178247619 | 3.533989209 | 0.00000349 | 0.000014 |
| RTN3 | 0.254987422 | 5.962542154 | 0.002687951 | 0.005998019 |
| RXRA | -0.286340567 | 5.734533275 | 0.008689153 | 0.017179095 |
| RYBP | -0.647675629 | 3.967521346 | 3.98E-09 | 0.000000027 |
| S100A11 | 0.96020326 | 7.956224788 | 0.0000231 | 0.0000792 |
| SBDS | -0.334599929 | 6.760033811 | 0.0000076 | 0.0000287 |
| SEC62 | -0.904348021 | 5.575656393 | 9.3E-33 | 9.95E-31 |
| SEC63 | -0.270957432 | 5.065061927 | 0.001144313 | 0.002774863 |
| SECTM1 | 0.661436822 | 4.263155576 | 0.002270317 | 0.005152191 |
| SEMA3B | 1.424705913 | 3.595146137 | 0.0000117 | 0.0000424 |
| SERPINE1 | -2.185184538 | 7.320384488 | 1.97E-28 | 1.38E-26 |
| SERPING1 | -1.223501127 | 10.86542099 | 4.57E-21 | 1.53E-19 |
| SFXN2 | -0.693358513 | 3.606415864 | 0.000000311 | 0.00000151 |
| SH2B2 | 0.747781075 | 2.986479831 | 0.013027878 | 0.024657856 |
| SH3PXD2A | 0.493106058 | 3.621171731 | 0.00647605 | 0.013211695 |
| SHKBP1 | 0.667524287 | 4.873918723 | 0.000000141 | 0.000000734 |
| SHMT1 | -1.282184995 | 6.708315972 | 1.49E-16 | 2.88E-15 |
| SHROOM1 | 0.422331552 | 5.580971409 | 0.001724625 | 0.004019593 |
| SIK3 | -0.491500609 | 3.71271128 | 0.000123473 | 0.000367782 |
| SIN3B | 0.532880929 | 3.593950007 | 0.004438204 | 0.009406659 |
| SIPA1L2 | 1.080758444 | 3.936633845 | 0.0000109 | 0.0000397 |
| SIRT1 | -0.684912841 | 3.757886341 | 9.21E-09 | 5.95E-08 |
| SIRT3 | -0.483313255 | 4.18114814 | 0.00000618 | 0.0000237 |
| SIRT6 | 0.61488647 | 4.238721857 | 0.00000559 | 0.0000216 |
| SLA | -0.734777903 | 3.029897266 | 0.000236141 | 0.000661493 |
| SLC12A9 | 0.614394162 | 3.888115347 | 0.0000592 | 0.000187191 |
| SLC15A3 | -0.42452016 | 3.817429793 | 0.007644399 | 0.015283874 |
| SLC20A2 | -0.417855973 | 4.729488336 | 0.001864915 | 0.004312573 |
| SLC22A23 | 0.721346179 | 3.970877047 | 0.0000105 | 0.0000387 |
| SLC25A37 | -0.574943181 | 3.386326481 | 0.00012307 | 0.000366848 |
| SLC2A3 | -0.894738162 | 3.490309722 | 0.00000806 | 0.0000302 |
| SLC38A1 | 0.991524622 | 3.882624727 | 0.000130037 | 0.000385302 |
| SLC43A2 | 0.565263535 | 3.284323082 | 0.013722718 | 0.025846916 |
| SLC45A4 | 1.65001358 | 3.02263304 | 0.00000631 | 0.0000242 |
| SLC6A12 | -1.142814294 | 4.985157634 | 2.39E-09 | 1.68E-08 |
| SLC6A6 | 0.757490012 | 3.117295611 | 0.020675449 | 0.037263822 |
| SLC7A1 | 0.8769272 | 3.064328561 | 0.004696318 | 0.009894617 |
| SMAP2 | -0.278190689 | 5.919386185 | 0.000611401 | 0.001569646 |
| SMARCB1 | 0.470348428 | 5.726613815 | 0.00000177 | 0.00000749 |
| SMARCE1 | 0.347502377 | 3.911415106 | 0.022252054 | 0.039719133 |
| SNRPA1 | 0.643434085 | 4.286760319 | 0.00000368 | 0.0000147 |
| SNRPF | 0.425516183 | 5.149507141 | 0.000733614 | 0.001850876 |
| SNX11 | 0.367886436 | 4.216668675 | 0.002992898 | 0.006603732 |
| SOCS3 | -2.282145667 | 5.660921162 | 5.27E-35 | 6.74E-33 |
| SOD2 | -0.578054019 | 6.89336743 | 0.000196259 | 0.000559492 |
| SPAG9 | -0.330251259 | 3.99996549 | 0.002483975 | 0.005578947 |
| SPRYD4 | -1.322512521 | 4.292689976 | 1.73E-31 | 1.64E-29 |
| SREBF1 | -0.436534311 | 6.042186126 | 0.003950962 | 0.008485479 |
| SSB | 0.370145972 | 5.000563229 | 0.000174418 | 0.000504054 |
| ST6GAL1 | -0.824694279 | 7.624290574 | 4.6E-12 | 4.82E-11 |
| STAT3 | -0.799916662 | 5.768203308 | 2.55E-17 | 5.4E-16 |
| STMN3 | 0.908890942 | 3.506896842 | 0.002981986 | 0.006580825 |
| STX11 | -1.244206314 | 3.082668429 | 1.61E-12 | 1.81E-11 |
| SULT1B1 | -0.846759971 | 3.649601143 | 0.000650641 | 0.001661115 |
| SWAP70 | 0.573998362 | 3.780675147 | 0.000565306 | 0.001459757 |
| SYNJ2BP | -0.485286569 | 4.14935312 | 0.00000387 | 0.0000154 |
| TAGLN2 | 0.877637181 | 8.60202206 | 1.26E-12 | 1.43E-11 |
| TARBP1 | 1.717634531 | 3.878995774 | 7.41E-18 | 1.68E-16 |
| TCEA3 | -0.370613867 | 6.713676471 | 0.001282044 | 0.003075816 |
| TCERG1 | 0.712724143 | 3.570004502 | 0.00018828 | 0.000539096 |
| TCF7 | 0.827344323 | 3.31630134 | 0.00174053 | 0.004050582 |
| TEF | 0.404920264 | 4.677929574 | 0.007212564 | 0.014516309 |
| TGFB1 | 0.605490618 | 5.082635209 | 0.005624211 | 0.011653839 |
| THBD | -1.20757469 | 3.628150722 | 2.15E-13 | 2.72E-12 |
| THEM4 | 0.638528104 | 3.910560563 | 0.000027 | 0.000091 |
| THRAP3 | -0.25339557 | 5.682964748 | 0.0000833 | 0.000255964 |
| TLR4 | -1.393431953 | 3.196841433 | 1.78E-19 | 4.96E-18 |
| TMC4 | 1.260094248 | 4.263300203 | 0.002032361 | 0.004659682 |
| TMEM120A | -0.277271031 | 6.863451159 | 0.007997327 | 0.015915194 |
| TMEM140 | -0.420156338 | 6.048698195 | 0.000160292 | 0.000466052 |
| TMEM164 | 1.245476659 | 3.452280088 | 2.34E-08 | 0.000000141 |
| TMEM209 | 0.496186999 | 3.868061577 | 0.001586994 | 0.003730323 |
| TMEM25 | -0.999193676 | 3.149625178 | 9.31E-09 | 6.01E-08 |
| TMEM41B | 0.224640974 | 4.932154019 | 0.017488641 | 0.032055194 |
| TMEM45B | 1.002482648 | 5.978763478 | 0.0000527 | 0.00016848 |
| TMEM88 | 0.491700972 | 3.706634909 | 0.007250906 | 0.014579299 |
| TMEM8B | 0.590504863 | 3.353195733 | 0.006056254 | 0.01245348 |
| TNFRSF1A | -0.82145514 | 6.601437437 | 1.62E-25 | 8.26E-24 |
| TNFSF10 | -0.462197137 | 6.393343464 | 0.003967051 | 0.008515616 |
| TOMM20 | 0.659462709 | 7.198977526 | 3.9E-12 | 4.14E-11 |
| TOP1MT | 0.584231036 | 4.489838371 | 0.000200345 | 0.000570484 |
| TOR1A | -0.531766208 | 4.865943145 | 1.86E-11 | 1.77E-10 |
| TP53I11 | 0.672509767 | 3.932845581 | 0.0001472 | 0.000431522 |
| TP53INP2 | -0.290890432 | 5.529284156 | 0.028342176 | 0.049224215 |
| TPST1 | -0.852918043 | 4.627329416 | 6.04E-11 | 5.39E-10 |
| TRANK1 | -0.925427194 | 3.427728159 | 1.18E-10 | 9.99E-10 |
| TRIB1 | -1.986413867 | 6.316719351 | 3.69E-61 | 2.08E-58 |
| TRIB2 | 0.846671334 | 4.300539399 | 0.0000562 | 0.000178641 |
| TRIM25 | 0.812549416 | 4.17215471 | 5.58E-09 | 3.72E-08 |
| TSC22D4 | 0.458531043 | 4.83412549 | 0.0000753 | 0.00023326 |
| TTLL4 | 1.382590114 | 3.644298652 | 0.000000034 | 0.000000199 |
| TUBA4A | 0.523011973 | 5.306415851 | 0.002363097 | 0.005343215 |
| TYK2 | 0.378399295 | 4.807295772 | 0.0000951 | 0.000288852 |
| UBE2C | 3.903318692 | 5.051907434 | 2.49E-33 | 2.74E-31 |
| UBL7 | 0.484239834 | 5.634133754 | 0.000000165 | 0.000000845 |
| UBN1 | 0.510891711 | 3.948037047 | 0.000662271 | 0.001687336 |
| UBR2 | -0.309681654 | 4.135199081 | 0.004022875 | 0.008620545 |
| UBXN2B | 0.432405754 | 4.35187918 | 0.002200753 | 0.00500622 |
| UPB1 | -0.937099275 | 6.298422991 | 0.000000344 | 0.00000166 |
| UPF3B | 0.897074845 | 3.893169423 | 2.94E-08 | 0.000000174 |
| USP14 | 0.485202574 | 4.770802262 | 0.00000169 | 0.00000718 |
| USP36 | 0.421003264 | 3.899028373 | 0.005871073 | 0.012108524 |
| VMO1 | -1.410557045 | 3.930750351 | 2.09E-22 | 8.08E-21 |
| VNN1 | -1.446248597 | 7.011362575 | 2.44E-09 | 1.71E-08 |
| VNN3 | -1.022260263 | 4.027406605 | 0.000000268 | 0.00000132 |
| VSIG4 | -1.832377377 | 4.126097558 | 1.59E-23 | 6.96E-22 |
| VTA1 | -0.340982825 | 3.903590986 | 0.002270973 | 0.005152738 |
| WDR75 | 0.338103523 | 4.053276593 | 0.016423093 | 0.030321434 |
| WIPF2 | 0.371253163 | 3.915259541 | 0.020870711 | 0.037583017 |
| XPA | -0.234436692 | 4.390089046 | 0.026161717 | 0.045828213 |
| XPR1 | 0.65042533 | 3.601220477 | 0.000522916 | 0.001360195 |
| YIPF3 | 0.513223341 | 6.998458466 | 5.07E-09 | 0.000000034 |
| YTHDC1 | -0.255911677 | 4.242554974 | 0.008441388 | 0.016733166 |
| Zbtb16 | -0.474345661 | 3.144197265 | 0.026871824 | 0.046946301 |
| ZBTB7B | 0.7505122 | 5.588585964 | 2.82E-09 | 1.96E-08 |
| ZCCHC14 | -0.428934185 | 4.25607154 | 0.0000297 | 0.0000992 |
| ZCCHC17 | 0.435801905 | 4.70326943 | 0.000031 | 0.000103287 |
| ZFAND3 | 0.263820326 | 5.857548178 | 0.003914436 | 0.008410104 |
| ZFP62 | 0.968503144 | 3.588142199 | 0.000000463 | 0.00000219 |
| ZFYVE1 | -0.279399436 | 3.886448696 | 0.022724531 | 0.040483917 |
| ZNF165 | 0.783948556 | 3.219602565 | 0.001850126 | 0.00428236 |
| ZNF195 | 0.53163444 | 3.291084537 | 0.023592232 | 0.041828717 |
| ZNF251 | 1.17845433 | 3.509523824 | 8.61E-09 | 5.59E-08 |
| ZNF302 | 0.389320611 | 3.817619805 | 0.015177336 | 0.028286381 |
| ZNF395 | -0.497891817 | 4.032875772 | 0.0000471 | 0.000152078 |
| ZNF552 | 0.652258087 | 3.214204317 | 0.00685107 | 0.013880991 |
| ZNF563 | 0.807014287 | 3.628148923 | 0.00000322 | 0.000013 |
| ZNF764 | 0.553529291 | 3.501077877 | 0.004056224 | 0.008683019 |
| ZNF787 | 0.408936547 | 5.308986199 | 0.000422575 | 0.001121747 |

**Supplementary Table 2.** Information on the 377 clinical samples of LIHC

| Id | futime | fustat | age | gender | grade | stage | T | M | N |
| --- | --- | --- | --- | --- | --- | --- | --- | --- | --- |
| TCGA-DD-A1EA | 2415 | 0 | 68 | MALE | G2 | Stage II | T2 | M0 | N0 |
| TCGA-KR-A7K0 | 65 | 1 | 65 | MALE | G1 | Stage I | T1 | M0 | N0 |
| TCGA-DD-A4NS | 2456 | 1 | 61 | FEMALE | G2 | Stage I | T1 | M0 | N0 |
| TCGA-CC-A5UC | 347 | 1 | 63 | MALE | G3 | Stage IIIA | T3 | M0 | N0 |
| TCGA-G3-AAV7 | 361 | 0 | 38 | MALE | G2 | Stage II | T2 | M0 | N0 |
| TCGA-DD-AAED | 763 | 0 | 51 | MALE | G3 | Stage I | T1 | M0 | N0 |
| TCGA-DD-AAE0 | 555 | 0 | 45 | FEMALE | G4 | Stage IIIA | T3a | M0 | N0 |
| TCGA-CC-A3M9 | 300 | 1 | 45 | MALE | G3 | Stage IIIA | T3 | M0 | N0 |
| TCGA-DD-A3A4 | 612 | 1 | 37 | MALE | G3 | Stage IIIA | T3 | M0 | N0 |
| TCGA-DD-AADB | 1242 | 0 | 51 | MALE | G4 | Stage I | T1 | M0 | N0 |
| TCGA-G3-A25V | 860 | 0 | 68 | MALE | G2 | Stage I | T1 | M0 | N0 |
| TCGA-DD-AAE7 | 644 | 0 | 72 | MALE | G2 | Stage I | T1 | M0 | N0 |
| TCGA-LG-A9QC | 425 | 0 | 48 | MALE | G2 | Stage I | T1 | M0 | NX |
| TCGA-DD-AAEH | 784 | 0 | 73 | MALE | G2 | Stage I | T1 | M0 | N0 |
| TCGA-DD-AAW1 | 1989 | 0 | 55 | MALE | G2 | Stage IIIA | T3 | M0 | N0 |
| TCGA-2Y-A9H9 | 697 | 0 | 70 | MALE | G2 | Stage I | T1 | MX | N0 |
| TCGA-5C-AAPD | 20 | 0 | 61 | MALE | G1 | Stage II | T2 | M0 | N0 |
| TCGA-EP-A2KA | 357 | 0 | 52 | FEMALE | G3 | Stage IIIA | T3a | MX | NX |
| TCGA-DD-A73B | 283 | 1 | 72 | FEMALE | G2 | Stage I | T1 | M0 | N0 |
| TCGA-UB-A7MD | 52 | 1 | 67 | MALE | G3 | Stage I | T1 | MX | N0 |
| TCGA-DD-A113 | 2425 | 0 | 55 | FEMALE | G3 | Stage II | T2 | M0 | N0 |
| TCGA-5C-A9VH | 322 | 0 | 70 | MALE | G2 | Stage I | T1 | M0 | N0 |
| TCGA-ED-A5KG | 482 | 0 | 60 | FEMALE | G2 | Stage II | T2 | M0 | N0 |
| TCGA-BC-A10U | 837 | 1 | 69 | MALE | G2 | unknow | T2 | MX | NX |
| TCGA-DD-A39V | 643 | 1 | 77 | MALE | G3 | Stage II | T2 | M0 | NX |
| TCGA-ED-A627 | 423 | 0 | 74 | MALE | G2 | Stage I | T1 | M0 | NX |
| TCGA-G3-AAV2 | 372 | 0 | 50 | MALE | G1 | Stage I | T1 | M0 | N0 |
| TCGA-ZS-A9CD | 1386 | 1 | 73 | MALE | G2 | Stage II | T2 | MX | NX |
| TCGA-GJ-A9DB | 67 | 1 | 68 | MALE | G2 | Stage I | T1 | MX | N0 |
| TCGA-G3-AAV4 | 27 | 1 | 83 | FEMALE | G1 | Stage I | T1 | M0 | N0 |
| TCGA-DD-AADP | 458 | 0 | 45 | MALE | G3 | Stage I | T1 | M0 | N0 |
| TCGA-DD-A4NL | 1711 | 0 | 46 | MALE | G1 | Stage I | T1 | M0 | N0 |
| TCGA-CC-5261 | 97 | 1 | 44 | MALE | G2 | Stage II | T2 | M0 | N0 |
| TCGA-UB-A7MC | 500 | 0 | 59 | MALE | G3 | Stage IIIA | T3a | MX | N0 |
| TCGA-CC-A7IF | 649 | 1 | 59 | MALE | G1 | Stage IIIA | T3 | M0 | N0 |
| TCGA-4R-AA8I | 262 | 1 | 66 | MALE | G2 | Stage II | T2 | MX | NX |
| TCGA-DD-A73C | 701 | 0 | 65 | FEMALE | G1 | Stage IIIA | T3a | M0 | N0 |
| TCGA-CC-5259 | 250 | 0 | 60 | FEMALE | G2 | Stage IIIC | T4 | M0 | N0 |
| TCGA-2Y-A9GW | 1271 | 1 | 64 | MALE | G2 | Stage I | T1 | MX | N0 |
| TCGA-BC-A10S | 1423 | 1 | 81 | MALE | G1 | unknow | T3 | MX | NX |
| TCGA-UB-A7ME | 486 | 0 | 51 | MALE | G2 | Stage I | T1 | MX | NX |
| TCGA-ED-A97K | 6 | 0 | 54 | MALE | G2 | Stage IIIA | T3a | M0 | N0 |
| TCGA-XR-A8TF | 693 | 1 | 74 | MALE | G1 | Stage I | T1 | MX | NX |
| TCGA-CC-A7II | 399 | 0 | 54 | MALE | G3 | Stage IIIA | T3 | M0 | N0 |
| TCGA-DD-AADQ | 436 | 0 | 59 | MALE | G3 | Stage II | T2 | M0 | N0 |
| TCGA-K7-A6G5 | 512 | 0 | 66 | MALE | G2 | Stage I | T1 | MX | N0 |
| TCGA-CC-5264 | 102 | 1 | 71 | MALE | G2 | Stage IIIA | T3 | M0 | N0 |
| TCGA-DD-A39Y | 171 | 1 | 67 | MALE | G3 | Stage I | T1 | M0 | NX |
| TCGA-ED-A8O6 | 56 | 1 | 50 | FEMALE | G3 | Stage IIIA | T3a | M0 | N0 |
| TCGA-DD-A1E9 | 2759 | 1 | 70 | MALE | G2 | Stage I | T1 | M0 | N0 |
| TCGA-DD-A1EC | 602 | 0 | 20 | FEMALE | G3 | Stage I | T1 | M0 | N0 |
| TCGA-DD-A115 | 2542 | 1 | 53 | MALE | G2 | Stage IIIA | T3 | M0 | N0 |
| TCGA-HP-A5MZ | 91 | 1 | 78 | MALE | G2 | Stage I | T1 | M0 | NX |
| TCGA-DD-A1EK | 558 | 1 | 64 | FEMALE | G2 | Stage IVB | T4 | M1 | N0 |
| TCGA-DD-AAE2 | 638 | 0 | 51 | MALE | G3 | Stage I | T1 | M0 | N0 |
| TCGA-CC-A5UE | 272 | 1 | 48 | MALE | G2 | Stage IIIB | T4 | M0 | N0 |
| TCGA-NI-A8LF | 606 | 0 | 74 | MALE | G3 | Stage I | T1 | MX | NX |
| TCGA-BW-A5NO | 20 | 0 | 50 | MALE | G2 | Stage IIIA | T3a | MX | NX |
| TCGA-CC-A5UD | 304 | 1 | 45 | MALE | G2 | Stage IIIA | T3 | M0 | N0 |
| TCGA-DD-A1EF | 394 | 1 | 57 | FEMALE | G3 | Stage I | T1 | M0 | N0 |
| TCGA-GJ-A3OU | 879 | 0 | 59 | MALE | G2 | Stage I | T1 | MX | NX |
| TCGA-DD-AAVU | 2202 | 0 | 46 | MALE | G2 | Stage II | T2 | M0 | N0 |
| TCGA-G3-A6UC | 671 | 0 | 65 | MALE | G2 | Stage IIIB | T3b | M0 | N0 |
| TCGA-CC-A9FU | 0 | 0 | 52 | FEMALE | G2 | Stage IIIA | T3a | M0 | N0 |
| TCGA-DD-AAD3 | 1295 | 0 | 43 | MALE | G2 | Stage I | T1 | M0 | N0 |
| TCGA-2Y-A9GU | 1939 | 0 | 55 | FEMALE | G2 | Stage I | T1 | MX | NX |
| TCGA-MI-A75G | 698 | 0 | 63 | MALE | G2 | Stage II | T2 | M0 | N0 |
| TCGA-FV-A4ZP | 2486 | 1 | 78 | MALE | G2 | Stage IIIA | T3 | M0 | NX |
| TCGA-PD-A5DF | 639 | 1 | 58 | FEMALE | G2 | Stage IIIB | T4 | M0 | N0 |
| TCGA-DD-A3A9 | 931 | 1 | 64 | FEMALE | G2 | Stage IVB | T4 | M1 | N0 |
| TCGA-DD-AACA | 2301 | 0 | 65 | MALE | G3 | Stage I | T1 | M0 | N0 |
| TCGA-DD-AAW2 | 1855 | 0 | 69 | MALE | G2 | Stage I | T1 | M0 | N0 |
| TCGA-DD-AADD | 1231 | 0 | 51 | MALE | G4 | Stage I | T1 | M0 | N0 |
| TCGA-XR-A8TG | 898 | 0 | 58 | MALE | G2 | Stage I | T1 | M0 | NX |
| TCGA-UB-AA0V | 314 | 0 | 69 | FEMALE | G1 | Stage I | unknow | MX | NX |
| TCGA-CC-A7IE | 217 | 1 | 57 | MALE | G2 | Stage IIIA | T3 | M0 | N0 |
| TCGA-BC-A69I | 387 | 0 | 69 | MALE | G1 | Stage I | T1 | M0 | N0 |
| TCGA-DD-AAE3 | 566 | 0 | 50 | MALE | G2 | Stage I | T1 | M0 | N0 |
| TCGA-CC-A1HT | 101 | 1 | 50 | MALE | G3 | Stage IIIA | T3 | M0 | N0 |
| TCGA-ZP-A9D1 | 21 | 0 | 56 | FEMALE | G2 | unknow | T1 | MX | NX |
| TCGA-EP-A3JL | 303 | 0 | 76 | MALE | G2 | Stage I | T1 | MX | NX |
| TCGA-CC-A7IL | 278 | 1 | 61 | MALE | G1 | Stage IIIA | T3 | M0 | N0 |
| TCGA-CC-5263 | 129 | 1 | 35 | MALE | G1 | Stage IIIA | T3 | M0 | N0 |
| TCGA-G3-A25X | 1779 | 0 | 73 | MALE | G3 | Stage II | T2 | M0 | N0 |
| TCGA-WQ-A9G7 | 30 | 0 | 71 | FEMALE | G3 | unknow | T3a | M0 | NX |
| TCGA-G3-A25Z | 655 | 0 | 58 | MALE | G2 | Stage I | T1 | M0 | N0 |
| TCGA-DD-AACX | 170 | 0 | 66 | MALE | G3 | Stage II | T2 | M0 | N0 |
| TCGA-DD-AAE6 | 141 | 0 | 59 | FEMALE | G2 | Stage I | T1 | M0 | N0 |
| TCGA-UB-AA0U | 327 | 0 | 60 | MALE | G2 | Stage II | T2 | MX | NX |
| TCGA-RG-A7D4 | 1098 | 0 | 69 | MALE | G2 | Stage II | T2 | M0 | N0 |
| TCGA-MI-A75I | 630 | 0 | 61 | MALE | G1 | unknow | T2 | MX | NX |
| TCGA-DD-AAVX | 1718 | 0 | 38 | MALE | G2 | Stage II | T2 | M0 | N0 |
| TCGA-EP-A2KC | 19 | 1 | 62 | MALE | G3 | Stage I | T1 | MX | NX |
| TCGA-DD-AADN | 898 | 0 | 59 | MALE | G4 | Stage I | T1 | MX | NX |
| TCGA-DD-A4NV | 2018 | 0 | 61 | MALE | G1 | Stage IIIA | T3 | M0 | N0 |
| TCGA-T1-A6J8 | 23 | 0 | 68 | MALE | G2 | unknow | T1 | M0 | NX |
| TCGA-DD-A4NK | 1210 | 1 | 80 | FEMALE | G2 | Stage IIIA | T3 | M0 | N0 |
| TCGA-CC-A7IJ | 382 | 0 | 56 | MALE | G3 | Stage II | T2 | M0 | N0 |
| TCGA-DD-AADR | 2028 | 0 | 58 | MALE | G3 | Stage I | T1 | M0 | N0 |
| TCGA-DD-A4NH | 690 | 0 | 65 | FEMALE | G3 | Stage IIIB | T3b | M0 | N0 |
| TCGA-BD-A2L6 | 1363 | 0 | 69 | MALE | G2 | unknow | T2 | MX | NX |
| TCGA-G3-A3CG | 673 | 0 | 80 | MALE | G2 | Stage I | T1 | M0 | N0 |
| TCGA-RC-A6M3 | 0 | 0 | 24 | MALE | G3 | Stage II | T2 | M0 | N0 |
| TCGA-DD-AACB | 2324 | 0 | 74 | FEMALE | G3 | Stage I | T1 | M0 | N0 |
| TCGA-BC-A10Y | 711 | 1 | 76 | MALE | G3 | unknow | T4 | MX | NX |
| TCGA-CC-A9FW | 248 | 0 | 68 | MALE | G2 | Stage IIIA | T3 | M0 | N0 |
| TCGA-DD-A1EL | 415 | 1 | 23 | MALE | G3 | Stage II | T2 | M0 | N0 |
| TCGA-DD-AAEE | 810 | 0 | 55 | MALE | G4 | Stage I | T1 | M0 | N0 |
| TCGA-BC-A10W | 91 | 1 | 50 | MALE | G3 | unknow | T4 | MX | NX |
| TCGA-CC-A8HU | 344 | 1 | 39 | FEMALE | G3 | Stage IIIA | T3 | M0 | N0 |
| TCGA-RC-A7SK | 472 | 0 | 59 | MALE | G3 | Stage I | T1 | M0 | N0 |
| TCGA-CC-5258 | 129 | 1 | 48 | MALE | G2 | Stage II | T2 | M0 | N0 |
| TCGA-UB-A7MF | 214 | 1 | 56 | MALE | G2 | Stage IIIA | T3a | MX | NX |
| TCGA-DD-AACL | 107 | 1 | 66 | FEMALE | G3 | Stage I | T1 | M0 | N0 |
| TCGA-BC-A10T | 837 | 1 | 76 | MALE | G1 | unknow | T4 | MX | NX |
| TCGA-DD-AACE | 2184 | 0 | 62 | MALE | G3 | Stage I | T1 | M0 | N0 |
| TCGA-BC-A110 | 2116 | 1 | 51 | FEMALE | G1 | unknow | T1 | MX | NX |
| TCGA-ED-A7PY | 390 | 0 | 20 | FEMALE | G3 | Stage II | T2 | M0 | NX |
| TCGA-DD-A4NR | 9 | 1 | 85 | FEMALE | G3 | Stage I | T1 | M0 | N0 |
| TCGA-BC-A8YO | 562 | 0 | 66 | FEMALE | G3 | Stage IIIC | T4 | M0 | N0 |
| TCGA-DD-AADU | 554 | 0 | 60 | MALE | G3 | Stage II | T2 | M0 | N0 |
| TCGA-BC-A10Z | 34 | 1 | 62 | FEMALE | G2 | Stage I | T1 | MX | N0 |
| TCGA-DD-AACK | 9 | 0 | 70 | MALE | G2 | Stage I | T1 | M0 | N0 |
| TCGA-DD-AAEK | 1067 | 0 | 51 | MALE | G3 | Stage II | T2 | M0 | N0 |
| TCGA-DD-AAW3 | 1633 | 0 | 69 | MALE | G2 | Stage I | T1 | M0 | N0 |
| TCGA-2Y-A9GZ | 848 | 1 | 82 | FEMALE | G2 | Stage II | T2 | MX | NX |
| TCGA-DD-A73F | 1085 | 0 | 77 | FEMALE | G1 | Stage I | T1 | M0 | N0 |
| TCGA-DD-AACS | 1804 | 0 | 39 | MALE | G3 | Stage I | T1 | M0 | N0 |
| TCGA-ED-A66Y | 296 | 1 | 51 | FEMALE | G3 | Stage IIIA | T3a | M0 | N0 |
| TCGA-G3-A3CJ | 594 | 0 | 52 | MALE | G2 | Stage II | T2 | M0 | N0 |
| TCGA-2Y-A9H2 | 1731 | 0 | 64 | FEMALE | G3 | Stage I | T1 | MX | N0 |
| TCGA-ED-A7PZ | 6 | 0 | 61 | MALE | G2 | Stage II | T2 | M0 | NX |
| TCGA-DD-AACI | 1618 | 0 | 69 | MALE | G3 | Stage II | T2 | M0 | N0 |
| TCGA-DD-A116 | 1622 | 1 | 68 | MALE | G3 | Stage IIIA | T3 | M0 | N0 |
| TCGA-DD-AAEG | 719 | 0 | 59 | FEMALE | G3 | Stage I | T1 | M0 | N0 |
| TCGA-DD-A114 | 1149 | 1 | 42 | MALE | G3 | Stage II | T2 | M0 | unknow |
| TCGA-ZS-A9CF | 2412 | 0 | 64 | MALE | G2 | Stage II | T2 | MX | NX |
| TCGA-RC-A6M6 | 9 | 0 | 75 | MALE | G3 | Stage II | T2 | M0 | NX |
| TCGA-5R-AA1C | 364 | 0 | 57 | MALE | G2 | Stage II | T2 | M0 | N0 |
| TCGA-CC-5262 | 103 | 1 | 67 | MALE | G1 | Stage IIIC | T4 | M0 | N0 |
| TCGA-DD-AACO | 1876 | 0 | 40 | MALE | G3 | Stage I | T1 | M0 | N0 |
| TCGA-ZP-A9CY | 782 | 0 | 66 | FEMALE | G1 | unknow | T1 | MX | NX |
| TCGA-ZP-A9D4 | 395 | 0 | 64 | FEMALE | G1 | unknow | T1 | MX | NX |
| TCGA-ZS-A9CE | 1241 | 0 | 79 | FEMALE | G1 | Stage II | T2 | MX | NX |
| TCGA-DD-AAVS | 1823 | 0 | 56 | MALE | G2 | Stage I | T1 | M0 | N0 |
| TCGA-XR-A8TC | 1339 | 0 | 43 | FEMALE | G2 | Stage I | T1 | MX | NX |
| TCGA-DD-A39W | 827 | 1 | 29 | FEMALE | G2 | Stage III | T3 | M0 | N0 |
| TCGA-BC-A69H | 444 | 0 | 64 | MALE | G3 | Stage II | T2 | M0 | NX |
| TCGA-DD-A4NA | 1008 | 0 | 67 | FEMALE | G3 | Stage IIIC | T2 | M0 | N1 |
| TCGA-MI-A75E | 507 | 0 | 61 | MALE | G2 | Stage IIIC | T4 | M0 | N0 |
| TCGA-DD-AAVZ | 1900 | 0 | 38 | MALE | G2 | Stage I | T1 | M0 | N0 |
| TCGA-G3-A5SL | 621 | 0 | 70 | MALE | G2 | Stage II | T2 | M0 | NX |
| TCGA-ED-A7XP | 400 | 0 | 53 | FEMALE | G3 | Stage II | T2 | M0 | N0 |
| TCGA-EP-A2KB | 334 | 0 | 46 | FEMALE | G2 | Stage I | T1 | MX | NX |
| TCGA-DD-A4NI | 561 | 0 | 67 | MALE | G2 | Stage II | T2 | M0 | NX |
| TCGA-CC-A9FV | 0 | 0 | 57 | MALE | G2 | Stage IIIA | T3 | M0 | N0 |
| TCGA-WQ-AB4B | 395 | 0 | 62 | MALE | G2 | Stage II | T2 | M0 | NX |
| TCGA-G3-AAV1 | 359 | 1 | 51 | MALE | G3 | Stage IIIC | T4 | M0 | N0 |
| TCGA-CC-A3MC | 363 | 0 | 54 | MALE | G2 | Stage IIIA | T3 | M0 | N0 |
| TCGA-G3-AAUZ | 480 | 0 | 48 | MALE | G2 | Stage I | T1 | M0 | N0 |
| TCGA-DD-AACM | 1769 | 0 | 48 | MALE | G3 | Stage II | T2 | M0 | N0 |
| TCGA-DD-A11D | 1560 | 1 | 57 | FEMALE | G2 | Stage I | T1 | M0 | N0 |
| TCGA-DD-AACF | 365 | 1 | 68 | MALE | G3 | Stage I | T1 | M0 | N0 |
| TCGA-BD-A3EP | 409 | 0 | 75 | FEMALE | G2 | Stage I | T1 | M0 | N0 |
| TCGA-ED-A4XI | 386 | 0 | 58 | MALE | G3 | Stage II | T2 | M0 | N0 |
| TCGA-DD-A11A | 79 | 0 | 67 | MALE | G3 | Stage I | T1 | M0 | N0 |
| TCGA-QA-A7B7 | 94 | 0 | 48 | MALE | G2 | Stage II | T2 | MX | NX |
| TCGA-DD-AADL | 636 | 0 | 58 | MALE | G4 | Stage I | T1 | M0 | N0 |
| TCGA-2Y-A9H6 | 357 | 0 | 68 | FEMALE | G2 | Stage I | T1 | MX | NX |
| TCGA-2Y-A9GY | 757 | 1 | 64 | FEMALE | G3 | Stage II | T2 | MX | NX |
| TCGA-DD-A73E | 44 | 0 | 66 | MALE | G1 | Stage I | T1 | M0 | N0 |
| TCGA-RC-A7SF | 579 | 0 | 66 | MALE | G2 | Stage I | T1 | M0 | N0 |
| TCGA-BC-A3KG | 498 | 0 | 68 | FEMALE | G3 | Stage II | T2 | M0 | N0 |
| TCGA-DD-AAVP | 2752 | 0 | 48 | MALE | G1 | Stage I | T1 | M0 | N0 |
| TCGA-ED-A459 | 408 | 0 | 47 | MALE | G2 | Stage II | T2 | M0 | N0 |
| TCGA-DD-AACW | 1424 | 0 | 43 | MALE | G3 | Stage I | T1 | M0 | N0 |
| TCGA-3K-AAZ8 | 396 | 0 | 65 | MALE | G1 | Stage IIIB | T3b | MX | NX |
| TCGA-G3-A7M5 | 447 | 0 | 76 | MALE | G2 | Stage I | T1 | MX | NX |
| TCGA-FV-A3R2 | 194 | 1 | 75 | MALE | unknow | Stage I | T1 | MX | NX |
| TCGA-BD-A3ER | 1115 | 0 | 62 | MALE | G2 | Stage II | T2 | MX | NX |
| TCGA-DD-AADY | 555 | 0 | 55 | FEMALE | G2 | Stage I | T1 | M0 | N0 |
| TCGA-DD-AACY | 1450 | 0 | 61 | MALE | G3 | Stage I | T1 | M0 | N0 |
| TCGA-DD-A1EI | 183 | 0 | 46 | MALE | G2 | Stage I | T1 | M0 | N0 |
| TCGA-2Y-A9HB | 260 | 0 | 66 | MALE | G2 | Stage I | T1 | MX | NX |
| TCGA-CC-A123 | 219 | 0 | 24 | FEMALE | G1 | Stage IIIA | T3 | M0 | N0 |
| TCGA-XR-A8TE | 925 | 0 | 16 | MALE | G1 | Stage IIIA | T3 | MX | N0 |
| TCGA-CC-A8HT | 140 | 1 | 74 | MALE | G2 | Stage IIIA | T3 | M0 | N0 |
| TCGA-G3-A5SM | 520 | 0 | 58 | MALE | G3 | Stage II | T2 | M0 | NX |
| TCGA-ES-A2HS | 688 | 1 | 80 | MALE | G2 | Stage I | T1 | MX | NX |
| TCGA-FV-A2QQ | 729 | 0 | 80 | MALE | G2 | Stage I | T1 | MX | N0 |
| TCGA-DD-AACU | 1567 | 0 | 59 | MALE | G3 | Stage I | T1 | M0 | N0 |
| TCGA-RC-A7SH | 468 | 0 | 42 | MALE | G3 | Stage II | T2 | M0 | N0 |
| TCGA-ZP-A9D2 | 743 | 0 | 51 | MALE | G2 | unknow | T2 | MX | NX |
| TCGA-DD-AACT | 1562 | 0 | 69 | FEMALE | G2 | Stage I | T1 | M0 | N0 |
| TCGA-DD-AADG | 1145 | 0 | 70 | MALE | G3 | Stage IIIA | T3a | M0 | N0 |
| TCGA-2Y-A9H0 | 3675 | 0 | 49 | MALE | G1 | Stage IIIA | T3 | M0 | N0 |
| TCGA-G3-AAV6 | 65 | 1 | 53 | FEMALE | G3 | Stage IIIA | T3a | M0 | N0 |
| TCGA-DD-A73D | 693 | 0 | 68 | FEMALE | G1 | Stage II | T2 | MX | NX |
| TCGA-FV-A2QR | 581 | 1 | 75 | MALE | G1 | Stage I | T1 | M0 | N0 |
| TCGA-DD-AADK | 1049 | 0 | 68 | FEMALE | G3 | Stage II | T2 | M0 | N0 |
| TCGA-DD-A4NE | 660 | 1 | 75 | FEMALE | G3 | Stage IIIA | T3a | M0 | N0 |
| TCGA-CC-A7IG | 299 | 1 | 47 | MALE | G2 | Stage II | T2 | M0 | N0 |
| TCGA-DD-A1EB | 2017 | 0 | 72 | FEMALE | G2 | Stage I | T1 | M0 | N0 |
| TCGA-DD-A118 | 3437 | 0 | 77 | FEMALE | G2 | Stage II | T2 | M0 | N0 |
| TCGA-5R-AAAM | 46 | 1 | 65 | FEMALE | G2 | Stage II | T2 | M0 | N0 |
| TCGA-DD-AAVQ | 2728 | 0 | 38 | MALE | G2 | Stage I | T1 | M0 | N0 |
| TCGA-G3-A25U | 1636 | 0 | 63 | FEMALE | G3 | Stage I | T1 | M0 | N0 |
| TCGA-G3-A7M8 | 430 | 0 | 31 | MALE | G1 | Stage I | T1 | MX | NX |
| TCGA-DD-A3A5 | 3125 | 1 | 66 | FEMALE | G2 | Stage III | T3 | M0 | N0 |
| TCGA-DD-AACZ | 171 | 1 | 63 | FEMALE | G4 | Stage I | T1 | M0 | N0 |
| TCGA-BC-A217 | 421 | 0 | 75 | FEMALE | G3 | Stage II | T2 | M0 | NX |
| TCGA-ED-A7XO | 427 | 0 | 29 | MALE | G2 | Stage IIIA | T3a | M0 | N0 |
| TCGA-FV-A495 | 1 | 0 | 51 | FEMALE | G2 | Stage II | T2 | M0 | NX |
| TCGA-DD-A1EH | 1495 | 0 | 23 | MALE | G3 | Stage III | T3 | M0 | N0 |
| TCGA-FV-A23B | 1852 | 1 | 70 | FEMALE | unknow | Stage II | T2 | M0 | N0 |
| TCGA-MI-A75C | 291 | 0 | 64 | MALE | G3 | Stage I | T1 | M0 | N0 |
| TCGA-DD-A4NP | 3104 | 0 | 32 | MALE | G3 | Stage I | T1 | M0 | N0 |
| TCGA-DD-AADF | 115 | 1 | 64 | FEMALE | G4 | Stage I | T1 | M0 | N0 |
| TCGA-DD-AAEA | 575 | 0 | 65 | MALE | G3 | Stage I | T1 | M0 | N0 |
| TCGA-EP-A3RK | 363 | 0 | 73 | MALE | G2 | Stage IIIA | T3a | MX | NX |
| TCGA-BC-4072 | 1490 | 1 | 74 | FEMALE | G3 | Stage IIIA | T3 | M0 | N0 |
| TCGA-FV-A3I0 | 848 | 0 | 76 | FEMALE | G2 | Stage II | T2 | M0 | NX |
| TCGA-UB-A7MB | 601 | 0 | 24 | MALE | G3 | Stage II | T2 | MX | NX |
| TCGA-ZP-A9CZ | 706 | 0 | 72 | MALE | G1 | unknow | T1 | MX | NX |
| TCGA-MI-A75H | 747 | 0 | 77 | MALE | unknow | unknow | unknow | MX | NX |
| TCGA-G3-A5SJ | 698 | 0 | 59 | MALE | G2 | Stage I | T1 | M0 | NX |
| TCGA-DD-AAD0 | 137 | 0 | 73 | FEMALE | G2 | Stage I | T1 | M0 | N0 |
| TCGA-ED-A82E | 408 | 0 | 60 | FEMALE | G2 | Stage IIIA | T3a | M0 | N0 |
| TCGA-BC-A5W4 | 547 | 1 | 69 | MALE | G3 | Stage IIIA | T3a | M0 | NX |
| TCGA-DD-AAEI | 1531 | 0 | 72 | MALE | G2 | Stage I | T1 | M0 | N0 |
| TCGA-2Y-A9GT | 1624 | 1 | 51 | MALE | G2 | Stage I | T1 | MX | NX |
| TCGA-XR-A8TD | 1030 | 0 | 49 | FEMALE | G3 | Stage IIIB | T3 | M0 | N0 |
| TCGA-2Y-A9H4 | 1452 | 0 | 68 | MALE | G2 | Stage I | T1 | MX | N0 |
| TCGA-DD-AACD | 381 | 1 | 48 | MALE | G4 | Stage I | T1 | M0 | N0 |
| TCGA-BC-A112 | 153 | 1 | 80 | MALE | G2 | unknow | T3 | MX | NX |
| TCGA-2Y-A9GV | 2532 | 1 | 54 | FEMALE | G1 | Stage I | T1 | MX | NX |
| TCGA-DD-AADJ | 1066 | 0 | 70 | FEMALE | G3 | Stage I | T1 | M0 | N0 |
| TCGA-CC-5260 | 87 | 1 | 61 | FEMALE | G1 | Stage IIIC | T4 | M0 | N0 |
| TCGA-LG-A6GG | 387 | 0 | 79 | FEMALE | G2 | Stage II | T2 | M0 | NX |
| TCGA-G3-AAV0 | 476 | 0 | 58 | MALE | G2 | Stage I | T1 | M0 | N0 |
| TCGA-DD-AACQ | 432 | 1 | 50 | MALE | G3 | Stage II | T2 | M0 | N0 |
| TCGA-KR-A7K7 | 407 | 0 | 61 | FEMALE | G1 | Stage II | T2 | M0 | N0 |
| TCGA-DD-A4NJ | 760 | 0 | 54 | FEMALE | G2 | Stage II | T2 | M0 | N0 |
| TCGA-DD-AAVV | 2455 | 0 | 56 | MALE | G3 | Stage II | T2 | M0 | N0 |
| TCGA-DD-AAC8 | 16 | 1 | 72 | MALE | G3 | Stage I | T1 | M0 | N0 |
| TCGA-G3-A25W | 935 | 0 | 79 | FEMALE | G2 | Stage IIIB | T3b | M0 | N0 |
| TCGA-WJ-A86L | 345 | 0 | 68 | FEMALE | G2 | Stage I | T1 | MX | NX |
| TCGA-CC-A3MB | 315 | 1 | 36 | MALE | G1 | Stage IIIA | T3 | M0 | N0 |
| TCGA-G3-A7M7 | 361 | 0 | 65 | MALE | G1 | Stage I | T1 | MX | NX |
| TCGA-DD-AADC | 425 | 1 | 53 | MALE | G3 | Stage I | T1 | M0 | N0 |
| TCGA-DD-AACC | 1685 | 1 | 61 | MALE | G2 | Stage I | T1 | M0 | N0 |
| TCGA-G3-A25T | 1553 | 0 | 45 | FEMALE | G2 | Stage IIIA | T3 | M0 | N0 |
| TCGA-G3-A3CH | 780 | 0 | 53 | MALE | G2 | Stage IIIA | T3a | M0 | N0 |
| TCGA-HP-A5N0 | 752 | 1 | 90 | FEMALE | unknow | unknow | TX | M0 | NX |
| TCGA-DD-A3A7 | 419 | 1 | 67 | MALE | G3 | Stage IIIB | T3b | M0 | N0 |
| TCGA-BC-4073 | 352 | 0 | 73 | MALE | G3 | Stage IIIA | T3 | MX | N0 |
| TCGA-2Y-A9HA | 36 | 1 | 70 | MALE | G2 | Stage II | T2 | MX | NX |
| TCGA-WX-AA44 | 615 | 0 | 64 | FEMALE | G3 | Stage I | T1 | MX | NX |
| TCGA-DD-A39Z | 601 | 1 | 43 | FEMALE | G2 | Stage II | T2 | M0 | NX |
| TCGA-DD-AAD5 | 1345 | 0 | 54 | MALE | G3 | Stage I | T1 | M0 | N0 |
| TCGA-2Y-A9H7 | 1168 | 0 | 81 | FEMALE | G2 | Stage I | T1 | MX | N0 |
| TCGA-DD-A1EJ | 1005 | 1 | 71 | FEMALE | G2 | Stage IIIC | T1 | M0 | N1 |
| TCGA-DD-A4NG | 802 | 1 | 77 | MALE | G2 | Stage IIIA | T3a | M0 | NX |
| TCGA-CC-A9FS | 211 | 0 | 55 | MALE | G2 | Stage II | T2 | M0 | N0 |
| TCGA-DD-A4NO | 2245 | 0 | 65 | MALE | G1 | Stage I | T1 | M0 | N0 |
| TCGA-DD-AAVY | 1970 | 0 | 56 | MALE | G2 | Stage IIIA | T3 | M0 | N0 |
| TCGA-G3-A25Y | 452 | 1 | 52 | FEMALE | G3 | Stage I | T1 | M0 | N0 |
| TCGA-BC-A3KF | 8 | 0 | 66 | FEMALE | G2 | Stage I | T1 | M0 | NX |
| TCGA-DD-A119 | 223 | 1 | 40 | MALE | G3 | Stage IV | T3a | M1 | N0 |
| TCGA-DD-AAVR | 2513 | 0 | 44 | MALE | G2 | Stage I | T1 | M0 | N0 |
| TCGA-UB-A7MA | 535 | 0 | 62 | FEMALE | G2 | Stage II | T2b | M0 | N0 |
| TCGA-ZS-A9CG | 341 | 0 | 55 | MALE | G2 | Stage II | T2 | MX | NX |
| TCGA-ES-A2HT | 438 | 1 | 54 | MALE | G2 | Stage I | T1 | MX | NX |
| TCGA-G3-A7M9 | 56 | 1 | 70 | MALE | G2 | Stage IIIB | T3b | MX | NX |
| TCGA-DD-AADI | 1085 | 0 | 43 | FEMALE | G3 | Stage I | T1 | M0 | N0 |
| TCGA-CC-A7IK | 262 | 1 | 59 | MALE | G3 | Stage IIIA | T3 | M0 | N0 |
| TCGA-DD-AADW | 587 | 0 | 48 | MALE | G3 | Stage I | T1 | M0 | N0 |
| TCGA-FV-A4ZQ | 12 | 0 | 52 | MALE | G2 | Stage I | T1 | M0 | NX |
| TCGA-DD-AADM | 12 | 1 | 58 | MALE | G3 | Stage II | T2 | M0 | N0 |
| TCGA-DD-AAW0 | 2015 | 0 | 54 | MALE | G2 | Stage I | T1 | M0 | N0 |
| TCGA-K7-AAU7 | 359 | 0 | 61 | MALE | G2 | Stage II | T2a | MX | NX |
| TCGA-ED-A8O5 | 406 | 0 | 59 | FEMALE | G3 | Stage IIIA | T3a | M0 | N0 |
| TCGA-DD-A73G | 3478 | 0 | 73 | FEMALE | G3 | Stage I | T1 | M0 | N0 |
| TCGA-DD-A11B | 14 | 1 | 73 | MALE | G2 | Stage I | T1 | M0 | N0 |
| TCGA-WX-AA46 | 756 | 0 | 61 | MALE | G1 | Stage II | T2 | MX | NX |
| TCGA-ED-A66X | 406 | 0 | 35 | MALE | G3 | Stage IIIA | T3a | M0 | N0 |
| TCGA-BC-A10X | 770 | 1 | 52 | FEMALE | G2 | Stage IIIA | T3a | MX | N0 |
| TCGA-DD-AADS | 474 | 0 | 63 | MALE | G2 | Stage I | T1 | M0 | N0 |
| TCGA-MR-A520 | 229 | 0 | 58 | MALE | G1 | Stage I | T1 | MX | NX |
| TCGA-LG-A9QD | 366 | 0 | 68 | MALE | G2 | Stage IIIA | T3a | M0 | N0 |
| TCGA-FV-A496 | 10 | 0 | 84 | FEMALE | G2 | Stage I | T1 | M0 | NX |
| TCGA-5C-A9VG | 328 | 0 | 58 | MALE | G2 | Stage II | T2 | M0 | N0 |
| TCGA-BC-A10R | 308 | 1 | 66 | FEMALE | G2 | unknow | T3 | MX | NX |
| TCGA-DD-AA3A | 410 | 1 | 81 | FEMALE | G4 | Stage I | T1 | MX | N0 |
| TCGA-DD-A73A | 728 | 0 | 71 | MALE | G2 | Stage I | T1 | M0 | N0 |
| TCGA-BW-A5NQ | 0 | 0 | 63 | MALE | G3 | Stage I | T1 | MX | NX |
| TCGA-DD-A4NB | 391 | 0 | 25 | MALE | G2 | Stage I | T1 | M0 | N0 |
| TCGA-DD-AADO | 453 | 0 | 55 | MALE | G3 | Stage I | T1 | M0 | N0 |
| TCGA-FV-A3R3 | 366 | 1 | 38 | FEMALE | G2 | Stage I | T1 | MX | NX |
| TCGA-RC-A7SB | 588 | 0 | 53 | MALE | G2 | Stage II | T2 | M0 | N0 |
| TCGA-DD-AAD2 | 658 | 0 | 66 | MALE | G2 | Stage I | T1 | M0 | N0 |
| TCGA-DD-A1EG | 1372 | 1 | 76 | MALE | G3 | Stage I | T1 | M0 | N0 |
| TCGA-G3-A3CI | 180 | 0 | 71 | MALE | G2 | Stage I | T1 | M0 | N0 |
| TCGA-DD-AAD1 | 564 | 0 | 51 | FEMALE | G4 | Stage I | T1 | M0 | N0 |
| TCGA-DD-A11C | 662 | 0 | 69 | MALE | G3 | Stage I | T1 | M0 | N0 |
| TCGA-WX-AA47 | 556 | 1 | 33 | FEMALE | G2 | Stage IIIA | T3a | MX | NX |
| TCGA-NI-A4U2 | 1791 | 1 | 71 | MALE | G1 | Stage IIIA | T3 | MX | NX |
| TCGA-2Y-A9H3 | 1516 | 0 | 45 | MALE | G1 | Stage II | T2 | MX | NX |
| TCGA-BC-A216 | 1351 | 0 | 62 | FEMALE | G2 | Stage IIIA | T3 | M0 | NX |
| TCGA-2Y-A9H5 | 555 | 1 | 59 | FEMALE | G3 | Stage I | T1 | MX | N0 |
| TCGA-EP-A12J | 330 | 0 | 62 | MALE | G1 | Stage I | T1 | MX | NX |
| TCGA-DD-AACP | 415 | 0 | 64 | MALE | G3 | Stage I | T1 | M0 | N0 |
| TCGA-DD-A3A2 | 2131 | 1 | 76 | FEMALE | G1 | Stage I | T1 | M0 | N0 |
| TCGA-DD-A3A0 | 785 | 1 | 70 | MALE | G2 | Stage I | T1 | M0 | NX |
| TCGA-DD-A4ND | 2232 | 0 | 56 | FEMALE | G3 | Stage I | T1 | M0 | N0 |
| TCGA-G3-AAV5 | 354 | 0 | 67 | MALE | G2 | Stage II | T2 | M0 | N0 |
| TCGA-DD-AAEB | 478 | 0 | 60 | MALE | G2 | Stage I | T1 | M0 | N0 |
| TCGA-2Y-A9H8 | 633 | 1 | 85 | FEMALE | G2 | unknow | T1 | MX | NX |
| TCGA-G3-A25S | 416 | 1 | 64 | MALE | G2 | Stage I | T1 | M0 | N0 |
| TCGA-RC-A6M5 | 15 | 0 | 20 | FEMALE | G2 | Stage IVA | T1 | M0 | N1 |
| TCGA-DD-AAE4 | 608 | 0 | 49 | FEMALE | G1 | Stage I | T1 | M0 | N0 |
| TCGA-DD-AACH | 195 | 1 | 69 | MALE | G3 | Stage II | T2 | M0 | N0 |
| TCGA-DD-AAD8 | 1219 | 0 | 73 | FEMALE | G2 | Stage I | T1 | M0 | N0 |
| TCGA-CC-A7IH | 365 | 0 | 58 | MALE | G1 | Stage IIIA | T3 | M0 | N0 |
| TCGA-DD-AAE9 | 722 | 0 | 69 | MALE | G3 | Stage I | T1 | M0 | N0 |
| TCGA-DD-AADA | 1233 | 0 | 66 | FEMALE | G3 | Stage I | T1 | M0 | N0 |
| TCGA-DD-A39X | 1694 | 1 | 78 | FEMALE | G2 | Stage I | T1 | M0 | NX |
| TCGA-DD-AADE | 1202 | 0 | 50 | MALE | G4 | Stage I | T1 | M0 | N0 |
| TCGA-K7-A5RG | 519 | 0 | 66 | MALE | G1 | Stage I | T1 | MX | NX |
| TCGA-DD-AAVW | 2317 | 0 | 35 | MALE | G2 | Stage I | T1 | M0 | N0 |
| TCGA-G3-A5SI | 768 | 1 | 44 | MALE | G2 | Stage II | T2 | M0 | N0 |
| TCGA-MR-A8JO | 330 | 0 | 34 | MALE | G3 | Stage I | T1 | MX | N0 |
| TCGA-DD-AAE1 | 552 | 0 | 52 | MALE | G3 | Stage I | T1 | M0 | N0 |
| TCGA-DD-A4NN | 899 | 1 | 56 | FEMALE | G3 | Stage I | T1 | M0 | N0 |
| TCGA-BW-A5NP | 0 | 0 | 26 | FEMALE | G3 | Stage IV | T2 | M1 | N0 |
| TCGA-CC-A8HV | 279 | 1 | 51 | FEMALE | G2 | Stage II | T2 | M0 | N0 |
| TCGA-G3-A3CK | 585 | 0 | 61 | MALE | G2 | Stage I | T1 | M0 | N0 |
| TCGA-EP-A26S | 237 | 0 | 70 | MALE | G2 | Stage I | T1 | MX | N0 |
| TCGA-2Y-A9GX | 2442 | 0 | 68 | MALE | G2 | Stage I | T1 | MX | NX |
| TCGA-RC-A7S9 | 640 | 0 | 47 | FEMALE | G3 | Stage I | T1 | M0 | N0 |
| TCGA-YA-A8S7 | 412 | 1 | 68 | MALE | G3 | Stage IIIA | T3a | MX | N0 |
| TCGA-DD-A3A3 | 535 | 1 | 45 | MALE | G2 | Stage I | T1 | M0 | N0 |
| TCGA-O8-A75V | 538 | 0 | 54 | MALE | G2 | Stage I | T1 | MX | NX |
| TCGA-ZP-A9D0 | 717 | 0 | 67 | FEMALE | G1 | unknow | T1 | MX | NX |
| TCGA-DD-A1EE | 349 | 1 | 73 | MALE | G3 | Stage IIIA | T3 | M0 | N0 |
| TCGA-DD-A3A6 | 3258 | 1 | 72 | FEMALE | G2 | Stage II | T2 | M0 | N0 |
| TCGA-RC-A6M4 | 22 | 0 | 74 | FEMALE | G2 | Stage IIIA | T3 | MX | NX |
| TCGA-DD-AAD6 | 672 | 0 | 66 | MALE | G3 | Stage IIIA | T3a | M0 | N0 |
| TCGA-K7-A5RF | 631 | 0 | 64 | MALE | G1 | Stage I | T1 | MX | NX |
| TCGA-CC-A8HS | 300 | 1 | 18 | MALE | G1 | Stage IIIC | T3 | M0 | N1 |
| TCGA-G3-A5SK | 744 | 0 | 58 | MALE | G1 | Stage I | T1 | M0 | NX |
| TCGA-DD-A4NQ | 373 | 1 | 60 | MALE | G3 | Stage II | T2 | M0 | N0 |
| TCGA-DD-A1ED | 2301 | 0 | 68 | MALE | G1 | Stage I | T1 | M0 | N0 |
| TCGA-G3-AAV3 | 412 | 0 | 58 | FEMALE | G2 | Stage II | T2 | M0 | N0 |
| TCGA-ZP-A9CV | 1088 | 1 | 59 | MALE | G1 | unknow | T1 | MX | NX |
| TCGA-DD-AACV | 1531 | 0 | 53 | MALE | G3 | Stage I | T1 | M0 | N0 |
| TCGA-DD-AACG | 469 | 1 | 52 | MALE | G4 | Stage II | T2 | M0 | N0 |
| TCGA-CC-A3MA | 303 | 1 | 61 | MALE | G2 | Stage IIIA | T3 | M0 | N0 |
| TCGA-2V-A95S |  | 0 | unknow | MALE | G3 | Stage II | T2 | MX | NX |
| TCGA-2Y-A9H1 | 1229 | 1 | 58 | MALE | G2 | Stage I | T1 | MX | NX |
| TCGA-DD-A4NF | 428 | 0 | 72 | MALE | G2 | Stage I | T1 | M0 | N0 |
| TCGA-DD-A3A1 | 233 | 1 | 65 | MALE | G2 | Stage IIIA | T3b | M0 | N0 |
| TCGA-KR-A7K2 | 657 | 0 | 64 | MALE | G1 | Stage I | T1 | M0 | N0 |
| TCGA-DD-AAC9 | 347 | 0 | 51 | MALE | G2 | Stage I | T1 | M0 | N0 |
| TCGA-DD-AACJ | 2102 | 0 | 75 | MALE | G2 | Stage II | T2 | M0 | N0 |
| TCGA-DD-AACN | 1302 | 0 | 32 | MALE | G3 | Stage I | T1 | M0 | N0 |
| TCGA-GJ-A6C0 | 31 | 1 | 75 | FEMALE | G2 | Stage II | T2 | MX | NX |
| TCGA-DD-AAE8 | 664 | 0 | 45 | MALE | G3 | Stage I | T1 | M0 | N0 |
| TCGA-DD-AADV | 574 | 0 | 50 | MALE | G3 | Stage I | T1 | M0 | N0 |
| TCGA-FV-A3I1 | 247 | 1 | 81 | FEMALE | G2 | Stage II | T2 | MX | N0 |
| TCGA-BC-A10Q | 1135 | 1 | 72 | FEMALE | unknow | unknow | T2 | MX | NX |
| TCGA-2Y-A9GS | 724 | 1 | 58 | MALE | G2 | unknow | T2 | MX | NX |
| TCGA-ED-A7PX | 6 | 0 | 48 | FEMALE | G3 | Stage II | T2 | M0 | NX |
| TCGA-DD-A3A8 | 11 | 1 | 75 | MALE | G2 | Stage II | T2 | M0 | N0 |
| TCGA-G3-A7M6 | 632 | 0 | 60 | FEMALE | G3 | Stage I | T1 | MX | NX |
| TCGA-KR-A7K8 | 906 | 0 | 57 | MALE | G1 | Stage I | T1 | M0 | N0 |
| TCGA-5R-AA1D | 337 | 0 | 17 | FEMALE | G3 | Stage IIIA | T3a | M0 | N0 |

**Supplementary Table 3.** Gene(*CSNK1D*, *CSNK1E* and *NPAS2*) expression profiles of 50 pairs of normal and tumour samples from the same patients.

| ID | CSNK1D | CSNK1E | NPAS2 | Type |
| --- | --- | --- | --- | --- |
| TCGA-DD-A3A4-11A-11R-A22L-07 | 6.786825 | 4.861762 | 0.8469471 | Normal |
| TCGA-BC-A10X-11A-11R-A131-07 | 2.967905 | 2.434108 | 0.3734686 | Normal |
| TCGA-BC-A10U-11A-11R-A131-07 | 6.254482 | 4.421511 | 0.6488311 | Normal |
| TCGA-DD-A11B-11A-11R-A131-07 | 4.2813 | 2.329953 | 0.4045968 | Normal |
| TCGA-DD-A39X-11A-11R-A213-07 | 3.272008 | 2.18899 | 0.293823 | Normal |
| TCGA-DD-A3A5-11A-11R-A22L-07 | 5.423726 | 1.866796 | 0.6406574 | Normal |
| TCGA-FV-A3I0-11A-11R-A22L-07 | 5.580588 | 3.128672 | 0.3761681 | Normal |
| TCGA-DD-A11A-11A-11R-A131-07 | 5.78008 | 2.707659 | 1.149496 | Normal |
| TCGA-DD-A39W-11A-11R-A213-07 | 3.100326 | 1.466508 | 0.1343296 | Normal |
| TCGA-DD-A1EC-11A-11R-A131-07 | 3.971439 | 2.657585 | 0.4280935 | Normal |
| TCGA-BC-A10Y-11A-11R-A131-07 | 4.801513 | 3.269734 | 0.4533046 | Normal |
| TCGA-BC-A10R-11A-11R-A131-07 | 6.806646 | 3.212199 | 1.205597 | Normal |
| TCGA-DD-A11D-11A-12R-A131-07 | 4.586691 | 3.125713 | 0.8499267 | Normal |
| TCGA-BC-A10W-11A-11R-A131-07 | 6.68727 | 3.86521 | 0.5547918 | Normal |
| TCGA-DD-A1EJ-11A-11R-A155-07 | 4.122732 | 3.148508 | 0.4449576 | Normal |
| TCGA-DD-A3A3-11A-11R-A22L-07 | 6.341228 | 3.332934 | 0.4913894 | Normal |
| TCGA-EP-A12J-11A-11R-A131-07 | 4.716111 | 3.02737 | 1.733312 | Normal |
| TCGA-DD-A3A8-11A-11R-A22L-07 | 4.609716 | 2.163566 | 0.2983224 | Normal |
| TCGA-DD-A119-11A-11R-A131-07 | 3.874417 | 1.81614 | 0.3730498 | Normal |
| TCGA-DD-A3A2-11A-11R-A213-07 | 2.812671 | 1.866829 | 1.021121 | Normal |
| TCGA-EP-A3RK-11A-11R-A22L-07 | 3.382574 | 1.912916 | 0.3493917 | Normal |
| TCGA-BC-A110-11A-11R-A131-07 | 6.6348 | 3.999661 | 0.7631256 | Normal |
| TCGA-DD-A116-11A-12R-A26B-07 | 3.235806 | 2.088198 | 0.2943724 | Normal |
| TCGA-DD-A3A6-11A-11R-A22L-07 | 3.121976 | 1.574241 | 0.2000863 | Normal |
| TCGA-BC-A10T-11A-11R-A131-07 | 4.112143 | 2.35283 | 0.9395465 | Normal |
| TCGA-DD-A1EG-11A-11R-A213-07 | 5.090309 | 2.908959 | 0.3530554 | Normal |
| TCGA-BC-A10Q-11A-11R-A131-07 | 3.977618 | 3.069182 | 1.914373 | Normal |
| TCGA-DD-A118-11A-11R-A131-07 | 4.883799 | 1.427575 | 1.420833 | Normal |
| TCGA-FV-A23B-11A-11R-A16W-07 | 5.016059 | 3.939016 | 0.7089196 | Normal |
| TCGA-EP-A26S-11A-12R-A16W-07 | 3.317016 | 1.420417 | 0.3100192 | Normal |
| TCGA-DD-A114-11A-12R-A131-07 | 5.044249 | 3.735678 | 0.4151834 | Normal |
| TCGA-FV-A3R2-11A-11R-A22L-07 | 5.306928 | 3.537692 | 0.4774828 | Normal |
| TCGA-DD-A1EH-11A-11R-A131-07 | 3.078717 | 2.011501 | 0.3719578 | Normal |
| TCGA-DD-A3A1-11A-11R-A213-07 | 3.54115 | 2.110854 | 0.4183127 | Normal |
| TCGA-DD-A1EB-11A-11R-A131-07 | 3.65444 | 2.713307 | 0.3026624 | Normal |
| TCGA-G3-A3CH-11A-11R-A22L-07 | 3.400191 | 2.010199 | 0.1579398 | Normal |
| TCGA-BD-A2L6-11A-21R-A213-07 | 5.765078 | 4.99517 | 0.5662052 | Normal |
| TCGA-DD-A39Z-11A-21R-A213-07 | 3.505999 | 2.17572 | 0.698245 | Normal |
| TCGA-ES-A2HT-11A-11R-A180-07 | 6.281591 | 3.976143 | 0.6062175 | Normal |
| TCGA-DD-A39V-11A-11R-A213-07 | 4.014708 | 2.377135 | 0.5808277 | Normal |
| TCGA-BC-A216-11A-11R-A155-07 | 2.942671 | 2.218638 | 1.060925 | Normal |
| TCGA-DD-A113-11A-12R-A131-07 | 4.972289 | 3.084844 | 1.765765 | Normal |
| TCGA-DD-A1EE-11A-11R-A131-07 | 4.158194 | 2.215434 | 1.570603 | Normal |
| TCGA-DD-A1EL-11A-11R-A155-07 | 4.319726 | 3.263244 | 0.3467692 | Normal |
| TCGA-FV-A2QR-11A-11R-A213-07 | 8.240958 | 6.974979 | 0.8561987 | Normal |
| TCGA-DD-A1EI-11A-11R-A131-07 | 3.348574 | 2.219899 | 0.4074718 | Normal |
| TCGA-BC-A10Z-11A-11R-A131-07 | 7.055348 | 6.824707 | 0.3639188 | Normal |
| TCGA-FV-A3I1-11A-11R-A22L-07 | 3.827291 | 2.470046 | 0.6647628 | Normal |
| TCGA-BD-A3EP-11A-12R-A22L-07 | 7.045571 | 3.574725 | 0.3489322 | Normal |
| TCGA-DD-A11C-11A-11R-A131-07 | 3.554568 | 2.177297 | 0.9815097 | Normal |
| TCGA-BC-A10T-01A-11R-A131-07 | 7.06014 | 4.708118 | 0.2647655 | Tumor |
| TCGA-DD-A3A2-01A-11R-A213-07 | 3.795242 | 3.770019 | 2.068983 | Tumor |
| TCGA-G3-A3CH-01A-11R-A22L-07 | 6.401132 | 6.152098 | 0.7198927 | Tumor |
| TCGA-DD-A1EI-01A-11R-A131-07 | 10.29144 | 9.531963 | 2.350419 | Tumor |
| TCGA-BC-A10Y-01A-11R-A131-07 | 12.79092 | 5.028779 | 0.6785344 | Tumor |
| TCGA-BC-A216-01A-11R-A155-07 | 8.999922 | 8.389054 | 3.18957 | Tumor |
| TCGA-DD-A39W-01A-11R-A213-07 | 8.774072 | 3.022379 | 0.5447689 | Tumor |
| TCGA-FV-A3R2-01A-11R-A22L-07 | 9.12125 | 7.074685 | 2.397875 | Tumor |
| TCGA-DD-A11B-01A-11R-A131-07 | 9.453359 | 3.974443 | 0.3566221 | Tumor |
| TCGA-DD-A1EL-01A-11R-A155-07 | 14.34799 | 10.51777 | 0.6052158 | Tumor |
| TCGA-DD-A3A3-01A-11R-A22L-07 | 5.875682 | 3.348454 | 0.3111008 | Tumor |
| TCGA-FV-A3I0-01A-11R-A22L-07 | 11.99866 | 27.97637 | 6.998379 | Tumor |
| TCGA-BC-A10R-01A-11R-A131-07 | 6.515967 | 11.81137 | 1.910444 | Tumor |
| TCGA-DD-A3A5-01A-11R-A22L-07 | 8.624143 | 6.978819 | 0.8739805 | Tumor |
| TCGA-DD-A11D-01A-11R-A131-07 | 6.093872 | 4.527248 | 2.109243 | Tumor |
| TCGA-ES-A2HT-01A-12R-A180-07 | 4.605796 | 2.232666 | 0.8145012 | Tumor |
| TCGA-DD-A1EB-01A-11R-A131-07 | 11.19743 | 5.770928 | 0.4466434 | Tumor |
| TCGA-FV-A23B-01A-11R-A16W-07 | 12.6982 | 8.271632 | 1.199642 | Tumor |
| TCGA-BC-A10W-01A-11R-A131-07 | 14.28751 | 6.364967 | 5.598503 | Tumor |
| TCGA-DD-A11C-01A-11R-A131-07 | 9.874856 | 4.528884 | 0.172294 | Tumor |
| TCGA-DD-A116-01A-11R-A131-07 | 10.22011 | 4.171181 | 1.480631 | Tumor |
| TCGA-DD-A39Z-01A-11R-A213-07 | 5.080009 | 6.051619 | 0.7522848 | Tumor |
| TCGA-EP-A3RK-01A-11R-A22L-07 | 10.80329 | 5.906511 | 0.6472099 | Tumor |
| TCGA-DD-A118-01A-11R-A131-07 | 10.71559 | 4.724401 | 0.7958616 | Tumor |
| TCGA-BC-A10Q-01A-11R-A131-07 | 15.4989 | 11.44269 | 2.443021 | Tumor |
| TCGA-DD-A113-01A-11R-A131-07 | 6.813736 | 3.792663 | 1.195586 | Tumor |
| TCGA-DD-A39X-01A-11R-A213-07 | 7.478129 | 4.039451 | 1.468483 | Tumor |
| TCGA-DD-A3A4-01A-11R-A22L-07 | 7.394099 | 3.564768 | 3.054171 | Tumor |
| TCGA-BC-A10X-01A-11R-A131-07 | 5.004669 | 5.052827 | 0.7798515 | Tumor |
| TCGA-DD-A1EH-01A-11R-A131-07 | 9.847202 | 9.018796 | 4.050741 | Tumor |
| TCGA-DD-A1EG-01A-11R-A213-07 | 8.336775 | 8.882002 | 1.342806 | Tumor |
| TCGA-BC-A10U-01A-11R-A131-07 | 17.00387 | 9.568827 | 2.462053 | Tumor |
| TCGA-FV-A3I1-01A-11R-A22L-07 | 10.35732 | 9.133986 | 2.017199 | Tumor |
| TCGA-DD-A119-01A-11R-A131-07 | 4.921314 | 3.590356 | 0.2517026 | Tumor |
| TCGA-BC-A10Z-01A-11R-A131-07 | 16.78344 | 6.479686 | 1.974111 | Tumor |
| TCGA-DD-A3A8-01A-11R-A22L-07 | 6.012817 | 2.527068 | 0.2897933 | Tumor |
| TCGA-EP-A26S-01A-11R-A16W-07 | 7.043432 | 3.398085 | 1.118118 | Tumor |
| TCGA-DD-A1EE-01A-11R-A131-07 | 8.509555 | 5.142578 | 1.871122 | Tumor |
| TCGA-BC-A110-01A-11R-A131-07 | 7.702327 | 6.972964 | 0.7380243 | Tumor |
| TCGA-BD-A2L6-01A-11R-A213-07 | 8.367098 | 3.119335 | 2.863574 | Tumor |
| TCGA-DD-A3A1-01A-11R-A213-07 | 11.86808 | 4.161481 | 1.529323 | Tumor |
| TCGA-DD-A1EC-01A-21R-A131-07 | 12.00732 | 7.459527 | 2.233609 | Tumor |
| TCGA-EP-A12J-01A-11R-A131-07 | 7.818429 | 2.473427 | 1.763025 | Tumor |
| TCGA-DD-A1EJ-01A-11R-A155-07 | 12.30389 | 13.72072 | 1.605104 | Tumor |
| TCGA-BD-A3EP-01A-11R-A22L-07 | 13.63531 | 8.349454 | 0.8983653 | Tumor |
| TCGA-DD-A39V-01A-11R-A213-07 | 6.201362 | 3.5757 | 0.6074523 | Tumor |
| TCGA-DD-A11A-01A-11R-A131-07 | 12.27023 | 3.773173 | 1.141807 | Tumor |
| TCGA-DD-A3A6-01A-11R-A22L-07 | 7.584609 | 9.968144 | 1.546555 | Tumor |
| TCGA-DD-A114-01A-11R-A131-07 | 19.20328 | 8.230695 | 4.803097 | Tumor |
| TCGA-FV-A2QR-01A-11R-A213-07 | 9.078807 | 6.57029 | 4.160961 | Tumor |

**Supplementary Table 4.** Univariate and multivariate analyses of prognostic factors（CSNK1D）

| Parameter | Univariate analysis | | | Multivariate analysis | | |
| --- | --- | --- | --- | --- | --- | --- |
|  | HR | 95% CI | P | HR | 95% CI | P |
| age | 1.01 | 0.99-1.02 | 0.591 | 1.01 | 0.99-1.03 | 0.456 |
| gender | 0.78 | 0.49-1.25 | 0.301 | 1.01 | 0.60-1.69 | 0.976 |
| grade | 1.02 | 0.75-1.39 | 0.914 | 1.06 | 0.75-1.48 | 0.753 |
| stage | 1.86 | 1.46-2.39 | <0.001 | 1.03 | 0.37-281 | 0.961 |
| T | 1.80 | 1.43-2.27 | <0.001 | 1.67 | 0.67-4.16 | 0.272 |
| M | 3.85 | 1.21-12.28 | 0.023 | 1.57 | 0.42-5.95 | 0.505 |
| N | 2.02 | 0.49-8.28 | 0.328 | 1.42 | 0.23-8.93 | 0.708 |
| CSNK1D | 2.28 | 1.47-3.54 | <0.001 | 2.06 | 1.26-3.37 | 0.004 |

HR: hazard ratio; CI: confidence interval.

**Supplementary Table 5.** Univariate and multivariate analyses of prognostic factors（CSNK1E）

| Parameter | Univariate analysis | | | Multivariate analysis | | |
| --- | --- | --- | --- | --- | --- | --- |
|  | HR | 95% CI | P | HR | 95% CI | P |
| age | 1.01 | 0.99-1.02 | 0.591 | 1.01 | 0.99-1.03 | 0.350 |
| gender | 0.78 | 0.49-1.25 | 0.301 | 1.12 | 0.66-1.90 | 0.674 |
| grade | 1.02 | 0.75-1.39 | 0.914 | 1.05 | 0.75-1.48 | 0.772 |
| stage | 1.86 | 1.46-2.39 | <0.001 | 1.11 | 0.42-2.96 | 0.835 |
| T | 1.80 | 1.43-2.27 | <0.001 | 1.63 | 0.67-3.97 | 0.279 |
| M | 3.85 | 1.21-12.28 | 0.023 | 1.17 | 0.31-4.36 | 0.818 |
| N | 2.02 | 0.49-8.28 | 0.328 | 1.43 | 0.22-9.13 | 0.706 |
| CSNK1E | 1.61 | 1.12-2.33 | 0.011 | 1.50 | 1.01-2.22 | 0.045 |

HR: hazard ratio; CI: confidence interval.

**Supplementary Table 6.** Univariate and multivariate analyses of prognostic factors（NPAS2）

| Parameter | Univariate analysis | | | Multivariate analysis | | |
| --- | --- | --- | --- | --- | --- | --- |
|  | HR | 95% CI | P | HR | 95% CI | P |
| age | 1.01 | 0.99-1.02 | 0.591 | 1.01 | 0.99-1.03 | 0.314 |
| gender | 0.78 | 0.49-1.25 | 0.301 | 1.12 | 0.66-1.90 | 0.674 |
| grade | 1.02 | 0.75-1.39 | 0.914 | 1.07 | 0.77-1.50 | 0.675 |
| stage | 1.86 | 1.46-2.39 | <0.001 | 1.09 | 0.41-2.86 | 0.865 |
| T | 1.80 | 1.43-2.27 | <0.001 | 1.61 | 0.67-3.87 | 0.285 |
| M | 3.85 | 1.21-12.28 | 0.023 | 1.25 | 0.33-4.66 | 0.742 |
| N | 2.02 | 0.49-8.28 | 0.328 | 2.34 | 0.37-14.58 | 0.364 |
| NPAS2 | 1.72 | 1.18-2.51 | 0.004 | 1.49 | 1.00-2.21 | 0.050 |

HR: hazard ratio; CI: confidence interval.

**Supplementary Table 7.** Raw data from the 235 patient samples of Cox analysis

| id | futime | fustat | age | gender | grade | stage | T | M | N |
| --- | --- | --- | --- | --- | --- | --- | --- | --- | --- |
| TCGA-DD-AAEK-01A-11R-A41C-07 | 1067 | 0 | 51 | MALE | G3 | Stage II | T2 | M0 | N0 |
| TCGA-DD-A4ND-01A-11R-A266-07 | 2232 | 0 | 56 | FEMALE | G3 | Stage I | T1 | M0 | N0 |
| TCGA-RC-A7S9-01A-11R-A33R-07 | 640 | 0 | 47 | FEMALE | G3 | Stage I | T1 | M0 | N0 |
| TCGA-DD-AADI-01A-11R-A41C-07 | 1085 | 0 | 43 | FEMALE | G3 | Stage I | T1 | M0 | N0 |
| TCGA-DD-A4NA-01A-11R-A266-07 | 1008 | 0 | 67 | FEMALE | G3 | Stage III | T2 | M0 | N1 |
| TCGA-RC-A7SH-01A-11R-A38B-07 | 468 | 0 | 42 | MALE | G3 | Stage II | T2 | M0 | N0 |
| TCGA-CC-A7IE-01A-21R-A38B-07 | 217 | 1 | 57 | MALE | G2 | Stage III | T3 | M0 | N0 |
| TCGA-DD-AAVS-01A-11R-A41C-07 | 1823 | 0 | 56 | MALE | G2 | Stage I | T1 | M0 | N0 |
| TCGA-DD-AAE7-01A-11R-A41C-07 | 644 | 0 | 72 | MALE | G2 | Stage I | T1 | M0 | N0 |
| TCGA-BC-A3KG-01A-11R-A213-07 | 498 | 0 | 68 | FEMALE | G3 | Stage II | T2 | M0 | N0 |
| TCGA-DD-A3A2-01A-11R-A213-07 | 2131 | 1 | 76 | FEMALE | G1 | Stage I | T1 | M0 | N0 |
| TCGA-DD-AAVR-01A-11R-A41C-07 | 2513 | 0 | 44 | MALE | G2 | Stage I | T1 | M0 | N0 |
| TCGA-CC-A7II-01A-11R-A33J-07 | 399 | 0 | 54 | MALE | G3 | Stage III | T3 | M0 | N0 |
| TCGA-DD-AACN-01A-11R-A41C-07 | 1302 | 0 | 32 | MALE | G3 | Stage I | T1 | M0 | N0 |
| TCGA-CC-A3MA-01A-11R-A213-07 | 303 | 1 | 61 | MALE | G2 | Stage III | T3 | M0 | N0 |
| TCGA-DD-AACQ-01A-11R-A41C-07 | 432 | 1 | 50 | MALE | G3 | Stage II | T2 | M0 | N0 |
| TCGA-RC-A7SF-01A-11R-A352-07 | 579 | 0 | 66 | MALE | G2 | Stage I | T1 | M0 | N0 |
| TCGA-DD-AADW-01A-11R-A39D-07 | 587 | 0 | 48 | MALE | G3 | Stage I | T1 | M0 | N0 |
| TCGA-G3-A3CH-01A-11R-A22L-07 | 780 | 0 | 53 | MALE | G2 | Stage III | T3 | M0 | N0 |
| TCGA-DD-A1EI-01A-11R-A131-07 | 183 | 0 | 46 | MALE | G2 | Stage I | T1 | M0 | N0 |
| TCGA-KR-A7K0-01A-12R-A33R-07 | 65 | 1 | 65 | MALE | G1 | Stage I | T1 | M0 | N0 |
| TCGA-ED-A8O5-01A-11R-A36F-07 | 406 | 0 | 59 | FEMALE | G3 | Stage III | T3 | M0 | N0 |
| TCGA-CC-A9FS-01A-11R-A37K-07 | 211 | 0 | 55 | MALE | G2 | Stage II | T2 | M0 | N0 |
| TCGA-DD-AACF-01A-11R-A41C-07 | 365 | 1 | 68 | MALE | G3 | Stage I | T1 | M0 | N0 |
| TCGA-DD-AADB-01A-11R-A41C-07 | 1242 | 0 | 51 | MALE | G4 | Stage I | T1 | M0 | N0 |
| TCGA-DD-AAVZ-01A-11R-A41C-07 | 1900 | 0 | 38 | MALE | G2 | Stage I | T1 | M0 | N0 |
| TCGA-DD-A1EF-01A-11R-A131-07 | 394 | 1 | 57 | FEMALE | G3 | Stage I | T1 | M0 | N0 |
| TCGA-DD-AADL-01A-11R-A41C-07 | 636 | 0 | 58 | MALE | G4 | Stage I | T1 | M0 | N0 |
| TCGA-KR-A7K8-01A-11R-A33J-07 | 906 | 0 | 57 | MALE | G1 | Stage I | T1 | M0 | N0 |
| TCGA-DD-AADK-01A-11R-A41C-07 | 1049 | 0 | 68 | FEMALE | G3 | Stage II | T2 | M0 | N0 |
| TCGA-DD-A39W-01A-11R-A213-07 | 827 | 1 | 29 | FEMALE | G2 | Stage III | T3 | M0 | N0 |
| TCGA-DD-A1EA-01A-11R-A131-07 | 2415 | 0 | 68 | MALE | G2 | Stage II | T2 | M0 | N0 |
| TCGA-G3-A3CJ-01A-11R-A213-07 | 594 | 0 | 52 | MALE | G2 | Stage II | T2 | M0 | N0 |
| TCGA-RC-A6M5-01A-11R-A32O-07 | 15 | 0 | 20 | FEMALE | G2 | Stage IV | T1 | M0 | N1 |
| TCGA-2Y-A9H0-01A-11R-A38B-07 | 3675 | 0 | 49 | MALE | G1 | Stage III | T3 | M0 | N0 |
| TCGA-DD-A11B-01A-11R-A131-07 | 14 | 1 | 73 | MALE | G2 | Stage I | T1 | M0 | N0 |
| TCGA-G3-A25V-01A-11R-A16W-07 | 860 | 0 | 68 | MALE | G2 | Stage I | T1 | M0 | N0 |
| TCGA-DD-AADQ-01A-11R-A41C-07 | 436 | 0 | 59 | MALE | G3 | Stage II | T2 | M0 | N0 |
| TCGA-CC-A8HS-01A-11R-A36F-07 | 300 | 1 | 18 | MALE | G1 | Stage III | T3 | M0 | N1 |
| TCGA-DD-A1EL-01A-11R-A155-07 | 415 | 1 | 23 | MALE | G3 | Stage II | T2 | M0 | N0 |
| TCGA-5R-AA1C-01A-11R-A41C-07 | 364 | 0 | 57 | MALE | G2 | Stage II | T2 | M0 | N0 |
| TCGA-ED-A8O6-01A-11R-A36F-07 | 56 | 1 | 50 | FEMALE | G3 | Stage III | T3 | M0 | N0 |
| TCGA-DD-AADD-01A-11R-A41C-07 | 1231 | 0 | 51 | MALE | G4 | Stage I | T1 | M0 | N0 |
| TCGA-CC-A9FV-01A-11R-A37K-07 | 0 | 0 | 57 | MALE | G2 | Stage III | T3 | M0 | N0 |
| TCGA-DD-AAEB-01A-11R-A41C-07 | 478 | 0 | 60 | MALE | G2 | Stage I | T1 | M0 | N0 |
| TCGA-PD-A5DF-01A-11R-A27V-07 | 639 | 1 | 58 | FEMALE | G2 | Stage III | T4 | M0 | N0 |
| TCGA-5R-AA1D-01A-11R-A38B-07 | 337 | 0 | 17 | FEMALE | G3 | Stage III | T3 | M0 | N0 |
| TCGA-DD-AACH-01A-11R-A41C-07 | 195 | 1 | 69 | MALE | G3 | Stage II | T2 | M0 | N0 |
| TCGA-CC-A8HV-01A-11R-A36F-07 | 279 | 1 | 51 | FEMALE | G2 | Stage II | T2 | M0 | N0 |
| TCGA-BW-A5NP-01A-11R-A27V-07 | 0 | 0 | 26 | FEMALE | G3 | Stage IV | T2 | M1 | N0 |
| TCGA-G3-AAV0-01A-11R-A37K-07 | 476 | 0 | 58 | MALE | G2 | Stage I | T1 | M0 | N0 |
| TCGA-DD-AAED-01A-12R-A41C-07 | 763 | 0 | 51 | MALE | G3 | Stage I | T1 | M0 | N0 |
| TCGA-DD-AADS-01A-11R-A41C-07 | 474 | 0 | 63 | MALE | G2 | Stage I | T1 | M0 | N0 |
| TCGA-DD-A3A3-01A-11R-A22L-07 | 535 | 1 | 45 | MALE | G2 | Stage I | T1 | M0 | N0 |
| TCGA-LG-A9QD-01A-11R-A38B-07 | 366 | 0 | 68 | MALE | G2 | Stage III | T3 | M0 | N0 |
| TCGA-ED-A7XP-01A-11R-A352-07 | 400 | 0 | 53 | FEMALE | G3 | Stage II | T2 | M0 | N0 |
| TCGA-DD-AAE2-01A-11R-A41C-07 | 638 | 0 | 51 | MALE | G3 | Stage I | T1 | M0 | N0 |
| TCGA-DD-AADF-01A-11R-A41C-07 | 115 | 1 | 64 | FEMALE | G4 | Stage I | T1 | M0 | N0 |
| TCGA-DD-AACW-01A-11R-A41C-07 | 1424 | 0 | 43 | MALE | G3 | Stage I | T1 | M0 | N0 |
| TCGA-CC-A8HU-01A-11R-A36F-07 | 344 | 1 | 39 | FEMALE | G3 | Stage III | T3 | M0 | N0 |
| TCGA-DD-AAD2-01A-11R-A41C-07 | 658 | 0 | 66 | MALE | G2 | Stage I | T1 | M0 | N0 |
| TCGA-CC-A1HT-01A-11R-A131-07 | 101 | 1 | 50 | MALE | G3 | Stage III | T3 | M0 | N0 |
| TCGA-ED-A7XO-01A-11R-A352-07 | 427 | 0 | 29 | MALE | G2 | Stage III | T3 | M0 | N0 |
| TCGA-CC-A5UC-01A-11R-A28V-07 | 347 | 1 | 63 | MALE | G3 | Stage III | T3 | M0 | N0 |
| TCGA-DD-AACJ-01A-11R-A41C-07 | 2102 | 0 | 75 | MALE | G2 | Stage II | T2 | M0 | N0 |
| TCGA-DD-A4NK-01A-11R-A28V-07 | 1210 | 1 | 80 | FEMALE | G2 | Stage III | T3 | M0 | N0 |
| TCGA-DD-A3A5-01A-11R-A22L-07 | 3125 | 1 | 66 | FEMALE | G2 | Stage III | T3 | M0 | N0 |
| TCGA-G3-AAUZ-01A-11R-A38B-07 | 480 | 0 | 48 | MALE | G2 | Stage I | T1 | M0 | N0 |
| TCGA-RC-A7SK-01A-11R-A352-07 | 472 | 0 | 59 | MALE | G3 | Stage I | T1 | M0 | N0 |
| TCGA-DD-AACI-01A-11R-A41C-07 | 1618 | 0 | 69 | MALE | G3 | Stage II | T2 | M0 | N0 |
| TCGA-DD-A4NH-01A-11R-A27V-07 | 690 | 0 | 65 | FEMALE | G3 | Stage III | T3 | M0 | N0 |
| TCGA-DD-A4NQ-01A-21R-A28V-07 | 373 | 1 | 60 | MALE | G3 | Stage II | T2 | M0 | N0 |
| TCGA-MI-A75E-01A-11R-A32O-07 | 507 | 0 | 61 | MALE | G2 | Stage III | T4 | M0 | N0 |
| TCGA-DD-A11D-01A-11R-A131-07 | 1560 | 1 | 57 | FEMALE | G2 | Stage I | T1 | M0 | N0 |
| TCGA-CC-5262-01A-01R-A131-07 | 103 | 1 | 67 | MALE | G1 | Stage III | T4 | M0 | N0 |
| TCGA-DD-AAW0-01A-11R-A41C-07 | 2015 | 0 | 54 | MALE | G2 | Stage I | T1 | M0 | N0 |
| TCGA-G3-A3CI-01A-11R-A213-07 | 180 | 0 | 71 | MALE | G2 | Stage I | T1 | M0 | N0 |
| TCGA-DD-AAVV-01A-11R-A41C-07 | 2455 | 0 | 56 | MALE | G3 | Stage II | T2 | M0 | N0 |
| TCGA-DD-A1EB-01A-11R-A131-07 | 2017 | 0 | 72 | FEMALE | G2 | Stage I | T1 | M0 | N0 |
| TCGA-DD-AAD1-01A-11R-A41C-07 | 564 | 0 | 51 | FEMALE | G4 | Stage I | T1 | M0 | N0 |
| TCGA-G3-A3CK-01A-11R-A213-07 | 585 | 0 | 61 | MALE | G2 | Stage I | T1 | M0 | N0 |
| TCGA-DD-AACA-02A-11R-A41C-07 | 2301 | 0 | 65 | MALE | G3 | Stage I | T1 | M0 | N0 |
| TCGA-5C-A9VH-01A-11R-A37K-07 | 322 | 0 | 70 | MALE | G2 | Stage I | T1 | M0 | N0 |
| TCGA-DD-AADR-01A-11R-A41C-07 | 2028 | 0 | 58 | MALE | G3 | Stage I | T1 | M0 | N0 |
| TCGA-DD-A4NO-01A-11R-A28V-07 | 2245 | 0 | 65 | MALE | G1 | Stage I | T1 | M0 | N0 |
| TCGA-DD-A11C-01A-11R-A131-07 | 662 | 0 | 69 | MALE | G3 | Stage I | T1 | M0 | N0 |
| TCGA-DD-AACV-01A-11R-A41C-07 | 1531 | 0 | 53 | MALE | G3 | Stage I | T1 | M0 | N0 |
| TCGA-CC-5264-01A-01R-A131-07 | 102 | 1 | 71 | MALE | G2 | Stage III | T3 | M0 | N0 |
| TCGA-DD-AACL-01A-11R-A41C-07 | 107 | 1 | 66 | FEMALE | G3 | Stage I | T1 | M0 | N0 |
| TCGA-DD-A116-01A-11R-A131-07 | 1622 | 1 | 68 | MALE | G3 | Stage III | T3 | M0 | N0 |
| TCGA-CC-A7IG-01A-11R-A33J-07 | 299 | 1 | 47 | MALE | G2 | Stage II | T2 | M0 | N0 |
| TCGA-DD-AAE9-01A-11R-A41C-07 | 722 | 0 | 69 | MALE | G3 | Stage I | T1 | M0 | N0 |
| TCGA-DD-AAVP-01A-11R-A41C-07 | 2752 | 0 | 48 | MALE | G1 | Stage I | T1 | M0 | N0 |
| TCGA-G3-AAV5-01A-11R-A37K-07 | 354 | 0 | 67 | MALE | G2 | Stage II | T2 | M0 | N0 |
| TCGA-CC-A3MB-01A-11R-A213-07 | 315 | 1 | 36 | MALE | G1 | Stage III | T3 | M0 | N0 |
| TCGA-CC-A7IF-01A-11R-A33J-07 | 649 | 1 | 59 | MALE | G1 | Stage III | T3 | M0 | N0 |
| TCGA-G3-A25S-01A-11R-A16W-07 | 416 | 1 | 64 | MALE | G2 | Stage I | T1 | M0 | N0 |
| TCGA-DD-AAW2-01A-11R-A41C-07 | 1855 | 0 | 69 | MALE | G2 | Stage I | T1 | M0 | N0 |
| TCGA-ED-A97K-01A-21R-A38B-07 | 6 | 0 | 54 | MALE | G2 | Stage III | T3 | M0 | N0 |
| TCGA-DD-AAVW-01A-11R-A41C-07 | 2317 | 0 | 35 | MALE | G2 | Stage I | T1 | M0 | N0 |
| TCGA-DD-A73F-01A-11R-A32O-07 | 1085 | 0 | 77 | FEMALE | G1 | Stage I | T1 | M0 | N0 |
| TCGA-DD-A4NN-01A-11R-A28V-07 | 899 | 1 | 56 | FEMALE | G3 | Stage I | T1 | M0 | N0 |
| TCGA-DD-AAEI-01A-11R-A41C-07 | 1531 | 0 | 72 | MALE | G2 | Stage I | T1 | M0 | N0 |
| TCGA-DD-AADO-01A-11R-A41C-07 | 453 | 0 | 55 | MALE | G3 | Stage I | T1 | M0 | N0 |
| TCGA-DD-AAVY-01A-11R-A41C-07 | 1970 | 0 | 56 | MALE | G2 | Stage III | T3 | M0 | N0 |
| TCGA-DD-A4NS-01A-11R-A311-07 | 2456 | 1 | 61 | FEMALE | G2 | Stage I | T1 | M0 | N0 |
| TCGA-DD-AAEG-01A-11R-A39D-07 | 719 | 0 | 59 | FEMALE | G3 | Stage I | T1 | M0 | N0 |
| TCGA-DD-AAD5-01A-11R-A41C-07 | 1345 | 0 | 54 | MALE | G3 | Stage I | T1 | M0 | N0 |
| TCGA-XR-A8TD-01A-12R-A39D-07 | 1030 | 0 | 49 | FEMALE | G3 | Stage III | T3 | M0 | N0 |
| TCGA-DD-AACY-01A-11R-A41C-07 | 1450 | 0 | 61 | MALE | G3 | Stage I | T1 | M0 | N0 |
| TCGA-CC-A9FU-01A-11R-A37K-07 | 0 | 0 | 52 | FEMALE | G2 | Stage III | T3 | M0 | N0 |
| TCGA-KR-A7K7-01A-11R-A33J-07 | 407 | 0 | 61 | FEMALE | G1 | Stage II | T2 | M0 | N0 |
| TCGA-G3-A25Y-01A-11R-A16W-07 | 452 | 1 | 52 | FEMALE | G3 | Stage I | T1 | M0 | N0 |
| TCGA-DD-AAW3-01A-11R-A41C-07 | 1633 | 0 | 69 | MALE | G2 | Stage I | T1 | M0 | N0 |
| TCGA-ED-A82E-01A-11R-A352-07 | 408 | 0 | 60 | FEMALE | G2 | Stage III | T3 | M0 | N0 |
| TCGA-DD-A4NP-01A-11R-A28V-07 | 3104 | 0 | 32 | MALE | G3 | Stage I | T1 | M0 | N0 |
| TCGA-G3-A6UC-01A-21R-A33J-07 | 671 | 0 | 65 | MALE | G2 | Stage III | T3 | M0 | N0 |
| TCGA-DD-AAW1-01A-11R-A41C-07 | 1989 | 0 | 55 | MALE | G2 | Stage III | T3 | M0 | N0 |
| TCGA-DD-A118-01A-11R-A131-07 | 3437 | 0 | 77 | FEMALE | G2 | Stage II | T2 | M0 | N0 |
| TCGA-DD-AADA-01A-11R-A41C-07 | 1233 | 0 | 66 | FEMALE | G3 | Stage I | T1 | M0 | N0 |
| TCGA-ED-A66Y-01A-11R-A311-07 | 296 | 1 | 51 | FEMALE | G3 | Stage III | T3 | M0 | N0 |
| TCGA-DD-AAE3-01A-11R-A41C-07 | 566 | 0 | 50 | MALE | G2 | Stage I | T1 | M0 | N0 |
| TCGA-G3-AAV6-01A-21R-A37K-07 | 65 | 1 | 53 | FEMALE | G3 | Stage III | T3 | M0 | N0 |
| TCGA-DD-A4NE-01A-11R-A27V-07 | 660 | 1 | 75 | FEMALE | G3 | Stage III | T3 | M0 | N0 |
| TCGA-DD-AADV-01A-11R-A39D-07 | 574 | 0 | 50 | MALE | G3 | Stage I | T1 | M0 | N0 |
| TCGA-DD-AACX-01A-11R-A41C-07 | 170 | 0 | 66 | MALE | G3 | Stage II | T2 | M0 | N0 |
| TCGA-DD-AACC-01A-11R-A41C-07 | 1685 | 1 | 61 | MALE | G2 | Stage I | T1 | M0 | N0 |
| TCGA-CC-A3MC-01A-11R-A22L-07 | 363 | 0 | 54 | MALE | G2 | Stage III | T3 | M0 | N0 |
| TCGA-DD-A73A-01A-12R-A32O-07 | 728 | 0 | 71 | MALE | G2 | Stage I | T1 | M0 | N0 |
| TCGA-DD-AADG-01A-11R-A41C-07 | 1145 | 0 | 70 | MALE | G3 | Stage III | T3 | M0 | N0 |
| TCGA-DD-A4NL-01A-11R-A28V-07 | 1711 | 0 | 46 | MALE | G1 | Stage I | T1 | M0 | N0 |
| TCGA-DD-A113-01A-11R-A131-07 | 2425 | 0 | 55 | FEMALE | G3 | Stage II | T2 | M0 | N0 |
| TCGA-RC-A6M3-01A-11R-A32O-07 | 0 | 0 | 24 | MALE | G3 | Stage II | T2 | M0 | N0 |
| TCGA-DD-AACU-01A-11R-A41C-07 | 1567 | 0 | 59 | MALE | G3 | Stage I | T1 | M0 | N0 |
| TCGA-DD-A3A4-01A-11R-A22L-07 | 612 | 1 | 37 | MALE | G3 | Stage III | T3 | M0 | N0 |
| TCGA-CC-A7IH-01A-11R-A33J-07 | 365 | 0 | 58 | MALE | G1 | Stage III | T3 | M0 | N0 |
| TCGA-DD-A1EH-01A-11R-A131-07 | 1495 | 0 | 23 | MALE | G3 | Stage III | T3 | M0 | N0 |
| TCGA-DD-AAEH-01A-11R-A41C-07 | 784 | 0 | 73 | MALE | G2 | Stage I | T1 | M0 | N0 |
| TCGA-DD-AAEE-01A-11R-A41C-07 | 810 | 0 | 55 | MALE | G4 | Stage I | T1 | M0 | N0 |
| TCGA-DD-A1EG-01A-11R-A213-07 | 1372 | 1 | 76 | MALE | G3 | Stage I | T1 | M0 | N0 |
| TCGA-DD-A115-01A-11R-A131-07 | 2542 | 1 | 53 | MALE | G2 | Stage III | T3 | M0 | N0 |
| TCGA-G3-AAV3-01A-11R-A37K-07 | 412 | 0 | 58 | FEMALE | G2 | Stage II | T2 | M0 | N0 |
| TCGA-UB-A7MA-01A-11R-A33R-07 | 535 | 0 | 62 | FEMALE | G2 | Stage II | T2 | M0 | N0 |
| TCGA-CC-A123-01A-11R-A131-07 | 219 | 0 | 24 | FEMALE | G1 | Stage III | T3 | M0 | N0 |
| TCGA-ED-A66X-01A-11R-A311-07 | 406 | 0 | 35 | MALE | G3 | Stage III | T3 | M0 | N0 |
| TCGA-DD-A1EK-01A-11R-A213-07 | 558 | 1 | 64 | FEMALE | G2 | Stage IV | T4 | M1 | N0 |
| TCGA-DD-AADM-01A-11R-A41C-07 | 12 | 1 | 58 | MALE | G3 | Stage II | T2 | M0 | N0 |
| TCGA-CC-A8HT-01A-11R-A36F-07 | 140 | 1 | 74 | MALE | G2 | Stage III | T3 | M0 | N0 |
| TCGA-DD-A4NJ-01A-11R-A27V-07 | 760 | 0 | 54 | FEMALE | G2 | Stage II | T2 | M0 | N0 |
| TCGA-CC-5258-01A-01R-A131-07 | 129 | 1 | 48 | MALE | G2 | Stage II | T2 | M0 | N0 |
| TCGA-G3-A25X-01A-11R-A16W-07 | 1779 | 0 | 73 | MALE | G3 | Stage II | T2 | M0 | N0 |
| TCGA-CC-A5UD-01A-11R-A28V-07 | 304 | 1 | 45 | MALE | G2 | Stage III | T3 | M0 | N0 |
| TCGA-DD-A119-01A-11R-A131-07 | 223 | 1 | 40 | MALE | G3 | Stage IV | T3 | M1 | N0 |
| TCGA-DD-AACB-01A-11R-A41C-07 | 2324 | 0 | 74 | FEMALE | G3 | Stage I | T1 | M0 | N0 |
| TCGA-DD-AAE4-01A-11R-A41C-07 | 608 | 0 | 49 | FEMALE | G1 | Stage I | T1 | M0 | N0 |
| TCGA-CC-A3M9-01A-11R-A213-07 | 300 | 1 | 45 | MALE | G3 | Stage III | T3 | M0 | N0 |
| TCGA-CC-A7IL-01A-11R-A33R-07 | 278 | 1 | 61 | MALE | G1 | Stage III | T3 | M0 | N0 |
| TCGA-DD-A3A8-01A-11R-A22L-07 | 11 | 1 | 75 | MALE | G2 | Stage II | T2 | M0 | N0 |
| TCGA-ED-A4XI-01A-11R-A266-07 | 386 | 0 | 58 | MALE | G3 | Stage II | T2 | M0 | N0 |
| TCGA-DD-AAVU-01A-11R-A41C-07 | 2202 | 0 | 46 | MALE | G2 | Stage II | T2 | M0 | N0 |
| TCGA-DD-AAD6-01A-11R-A41C-07 | 672 | 0 | 66 | MALE | G3 | Stage III | T3 | M0 | N0 |
| TCGA-DD-AAVX-01A-11R-A41C-07 | 1718 | 0 | 38 | MALE | G2 | Stage II | T2 | M0 | N0 |
| TCGA-DD-AADC-01A-11R-A41C-07 | 425 | 1 | 53 | MALE | G3 | Stage I | T1 | M0 | N0 |
| TCGA-DD-A3A7-01A-11R-A22L-07 | 419 | 1 | 67 | MALE | G3 | Stage III | T3 | M0 | N0 |
| TCGA-DD-AACE-01A-11R-A41C-07 | 2184 | 0 | 62 | MALE | G3 | Stage I | T1 | M0 | N0 |
| TCGA-DD-A4NB-01A-12R-A266-07 | 391 | 0 | 25 | MALE | G2 | Stage I | T1 | M0 | N0 |
| TCGA-G3-A5SI-01A-31R-A27V-07 | 768 | 1 | 44 | MALE | G2 | Stage II | T2 | M0 | N0 |
| TCGA-DD-AAE6-01A-11R-A41C-07 | 141 | 0 | 59 | FEMALE | G2 | Stage I | T1 | M0 | N0 |
| TCGA-DD-AACG-01A-11R-A41C-07 | 469 | 1 | 52 | MALE | G4 | Stage II | T2 | M0 | N0 |
| TCGA-5C-A9VG-01A-11R-A37K-07 | 328 | 0 | 58 | MALE | G2 | Stage II | T2 | M0 | N0 |
| TCGA-DD-A1EE-01A-11R-A131-07 | 349 | 1 | 73 | MALE | G3 | Stage III | T3 | M0 | N0 |
| TCGA-G3-A25U-01A-11R-A16W-07 | 1636 | 0 | 63 | FEMALE | G3 | Stage I | T1 | M0 | N0 |
| TCGA-G3-A3CG-01A-11R-A213-07 | 673 | 0 | 80 | MALE | G2 | Stage I | T1 | M0 | N0 |
| TCGA-CC-5260-01A-01R-A131-07 | 87 | 1 | 61 | FEMALE | G1 | Stage III | T4 | M0 | N0 |
| TCGA-CC-A7IJ-01A-11R-A33R-07 | 382 | 0 | 56 | MALE | G3 | Stage II | T2 | M0 | N0 |
| TCGA-DD-AADU-01A-11R-A41C-07 | 554 | 0 | 60 | MALE | G3 | Stage II | T2 | M0 | N0 |
| TCGA-DD-AAE1-01A-11R-A41C-07 | 552 | 0 | 52 | MALE | G3 | Stage I | T1 | M0 | N0 |
| TCGA-DD-A3A9-01A-11R-A266-07 | 931 | 1 | 64 | FEMALE | G2 | Stage IV | T4 | M1 | N0 |
| TCGA-KR-A7K2-01A-12R-A33R-07 | 657 | 0 | 64 | MALE | G1 | Stage I | T1 | M0 | N0 |
| TCGA-DD-A3A1-01A-11R-A213-07 | 233 | 1 | 65 | MALE | G2 | Stage III | T3 | M0 | N0 |
| TCGA-DD-A4NR-01A-11R-A311-07 | 9 | 1 | 85 | FEMALE | G3 | Stage I | T1 | M0 | N0 |
| TCGA-DD-AAC9-01A-11R-A41C-07 | 347 | 0 | 51 | MALE | G2 | Stage I | T1 | M0 | N0 |
| TCGA-CC-5259-01A-31R-A213-07 | 250 | 0 | 60 | FEMALE | G2 | Stage III | T4 | M0 | N0 |
| TCGA-DD-A1EC-01A-21R-A131-07 | 602 | 0 | 20 | FEMALE | G3 | Stage I | T1 | M0 | N0 |
| TCGA-DD-AAD3-01A-11R-A41C-07 | 1295 | 0 | 43 | MALE | G2 | Stage I | T1 | M0 | N0 |
| TCGA-DD-AACS-01A-11R-A41C-07 | 1804 | 0 | 39 | MALE | G3 | Stage I | T1 | M0 | N0 |
| TCGA-BC-A8YO-01A-11R-A37K-07 | 562 | 0 | 66 | FEMALE | G3 | Stage III | T4 | M0 | N0 |
| TCGA-CC-A9FW-01A-11R-A37K-07 | 248 | 0 | 68 | MALE | G2 | Stage III | T3 | M0 | N0 |
| TCGA-DD-A1ED-01A-11R-A155-07 | 2301 | 0 | 68 | MALE | G1 | Stage I | T1 | M0 | N0 |
| TCGA-DD-A1EJ-01A-11R-A155-07 | 1005 | 1 | 71 | FEMALE | G2 | Stage III | T1 | M0 | N1 |
| TCGA-G3-AAV2-01A-11R-A37K-07 | 372 | 0 | 50 | MALE | G1 | Stage I | T1 | M0 | N0 |
| TCGA-DD-AADY-01A-11R-A41C-07 | 555 | 0 | 55 | FEMALE | G2 | Stage I | T1 | M0 | N0 |
| TCGA-CC-5263-01A-01R-A131-07 | 129 | 1 | 35 | MALE | G1 | Stage III | T3 | M0 | N0 |
| TCGA-DD-AADP-01A-11R-A39D-07 | 458 | 0 | 45 | MALE | G3 | Stage I | T1 | M0 | N0 |
| TCGA-BC-A69I-01A-11R-A311-07 | 387 | 0 | 69 | MALE | G1 | Stage I | T1 | M0 | N0 |
| TCGA-G3-AAV1-01A-11R-A38B-07 | 359 | 1 | 51 | MALE | G3 | Stage III | T4 | M0 | N0 |
| TCGA-DD-AAE0-01A-11R-A41C-07 | 555 | 0 | 45 | FEMALE | G4 | Stage III | T3 | M0 | N0 |
| TCGA-DD-A73B-01A-12R-A32O-07 | 283 | 1 | 72 | FEMALE | G2 | Stage I | T1 | M0 | N0 |
| TCGA-RC-A7SB-01A-21R-A352-07 | 588 | 0 | 53 | MALE | G2 | Stage II | T2 | M0 | N0 |
| TCGA-CC-A5UE-01A-11R-A28V-07 | 272 | 1 | 48 | MALE | G2 | Stage III | T4 | M0 | N0 |
| TCGA-DD-AACZ-01A-11R-A41C-07 | 171 | 1 | 63 | FEMALE | G4 | Stage I | T1 | M0 | N0 |
| TCGA-DD-AAVQ-01A-11R-A41C-07 | 2728 | 0 | 38 | MALE | G2 | Stage I | T1 | M0 | N0 |
| TCGA-BD-A3EP-01A-11R-A22L-07 | 409 | 0 | 75 | FEMALE | G2 | Stage I | T1 | M0 | N0 |
| TCGA-DD-A4NF-01A-11R-A27V-07 | 428 | 0 | 72 | MALE | G2 | Stage I | T1 | M0 | N0 |
| TCGA-DD-A11A-01A-11R-A131-07 | 79 | 0 | 67 | MALE | G3 | Stage I | T1 | M0 | N0 |
| TCGA-DD-AAD0-01A-11R-A41C-07 | 137 | 0 | 73 | FEMALE | G2 | Stage I | T1 | M0 | N0 |
| TCGA-DD-A3A6-01A-11R-A22L-07 | 3258 | 1 | 72 | FEMALE | G2 | Stage II | T2 | M0 | N0 |
| TCGA-5C-AAPD-01A-21R-A39D-07 | 20 | 0 | 61 | MALE | G1 | Stage II | T2 | M0 | N0 |
| TCGA-DD-AACK-01A-11R-A41C-07 | 9 | 0 | 70 | MALE | G2 | Stage I | T1 | M0 | N0 |
| TCGA-MI-A75C-01A-11R-A32O-07 | 291 | 0 | 64 | MALE | G3 | Stage I | T1 | M0 | N0 |
| TCGA-DD-AACP-01A-11R-A41C-07 | 415 | 0 | 64 | MALE | G3 | Stage I | T1 | M0 | N0 |
| TCGA-DD-AACT-01A-11R-A41C-07 | 1562 | 0 | 69 | FEMALE | G2 | Stage I | T1 | M0 | N0 |
| TCGA-DD-A73E-01A-12R-A32O-07 | 44 | 0 | 66 | MALE | G1 | Stage I | T1 | M0 | N0 |
| TCGA-BC-4072-01B-11R-A155-07 | 1490 | 1 | 74 | FEMALE | G3 | Stage III | T3 | M0 | N0 |
| TCGA-DD-AAC8-01A-11R-A41C-07 | 16 | 1 | 72 | MALE | G3 | Stage I | T1 | M0 | N0 |
| TCGA-DD-AADJ-01A-11R-A41C-07 | 1066 | 0 | 70 | FEMALE | G3 | Stage I | T1 | M0 | N0 |
| TCGA-ED-A5KG-01A-11R-A27V-07 | 482 | 0 | 60 | FEMALE | G2 | Stage II | T2 | M0 | N0 |
| TCGA-DD-AACO-01A-11R-A41C-07 | 1876 | 0 | 40 | MALE | G3 | Stage I | T1 | M0 | N0 |
| TCGA-G3-A25Z-01A-11R-A16W-07 | 655 | 0 | 58 | MALE | G2 | Stage I | T1 | M0 | N0 |
| TCGA-DD-A4NV-01A-11R-A311-07 | 2018 | 0 | 61 | MALE | G1 | Stage III | T3 | M0 | N0 |
| TCGA-FV-A2QR-01A-11R-A213-07 | 581 | 1 | 75 | MALE | G1 | Stage I | T1 | M0 | N0 |
| TCGA-G3-AAV7-01A-11R-A38B-07 | 361 | 0 | 38 | MALE | G2 | Stage II | T2 | M0 | N0 |
| TCGA-CC-5261-01A-01R-A131-07 | 97 | 1 | 44 | MALE | G2 | Stage II | T2 | M0 | N0 |
| TCGA-G3-A25T-01A-11R-A16W-07 | 1553 | 0 | 45 | FEMALE | G2 | Stage III | T3 | M0 | N0 |
| TCGA-DD-AAEA-01A-11R-A41C-07 | 575 | 0 | 65 | MALE | G3 | Stage I | T1 | M0 | N0 |
| TCGA-RG-A7D4-01A-12R-A33R-07 | 1098 | 0 | 69 | MALE | G2 | Stage II | T2 | M0 | N0 |
| TCGA-DD-AACD-01A-11R-A41C-07 | 381 | 1 | 48 | MALE | G4 | Stage I | T1 | M0 | N0 |
| TCGA-DD-AAD8-01A-11R-A41C-07 | 1219 | 0 | 73 | FEMALE | G2 | Stage I | T1 | M0 | N0 |
| TCGA-5R-AAAM-01A-12R-A41C-07 | 46 | 1 | 65 | FEMALE | G2 | Stage II | T2 | M0 | N0 |
| TCGA-DD-A73G-01A-22R-A32O-07 | 3478 | 0 | 73 | FEMALE | G3 | Stage I | T1 | M0 | N0 |
| TCGA-G3-AAV4-01A-11R-A38B-07 | 27 | 1 | 83 | FEMALE | G1 | Stage I | T1 | M0 | N0 |
| TCGA-ED-A459-01A-11R-A266-07 | 408 | 0 | 47 | MALE | G2 | Stage II | T2 | M0 | N0 |
| TCGA-DD-A73C-01A-12R-A33J-07 | 701 | 0 | 65 | FEMALE | G1 | Stage III | T3 | M0 | N0 |
| TCGA-MI-A75G-01A-11R-A32O-07 | 698 | 0 | 63 | MALE | G2 | Stage II | T2 | M0 | N0 |
| TCGA-CC-A7IK-01A-12R-A33R-07 | 262 | 1 | 59 | MALE | G3 | Stage III | T3 | M0 | N0 |
